# Supplementary material for: Exploring emergency department 4-hour target performance and cancelled elective operations: a regression analysis of routinely collected and openly reported NHS trust data
Source: BMJ Open. 2018 May 24;8(5):e020296. doi: 10.1136/bmjopen-2017-020296 (PMC5988090; doi:10.1136/bmjopen-2017-020296)
Supplement: Supplementary data [file bmjopen-2017-020296supp001.pdf]

Appendix A: Plotted outputs for sample regression case in 2016. All other models can be built, and similar plots produced, by using the data and code provided in the additional supplementary files.

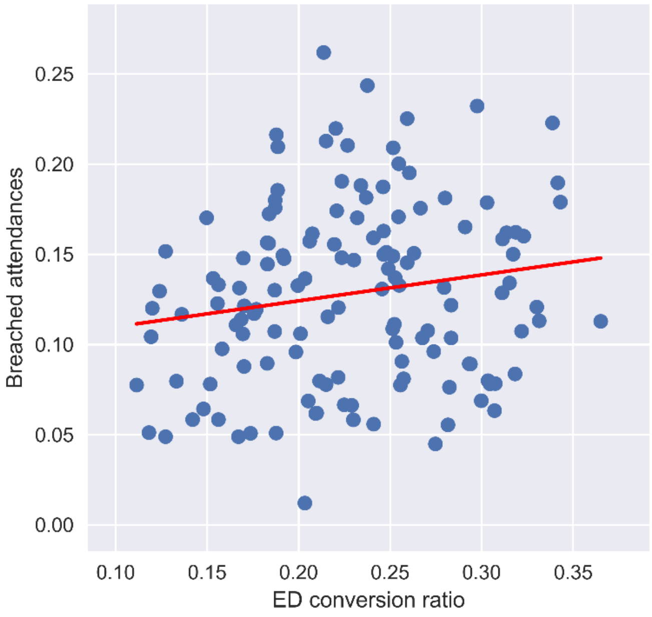

Figure A-1: Scatter plot of 'Breached attendances' and 'ED conversion ratio' with univariate linear regression model.

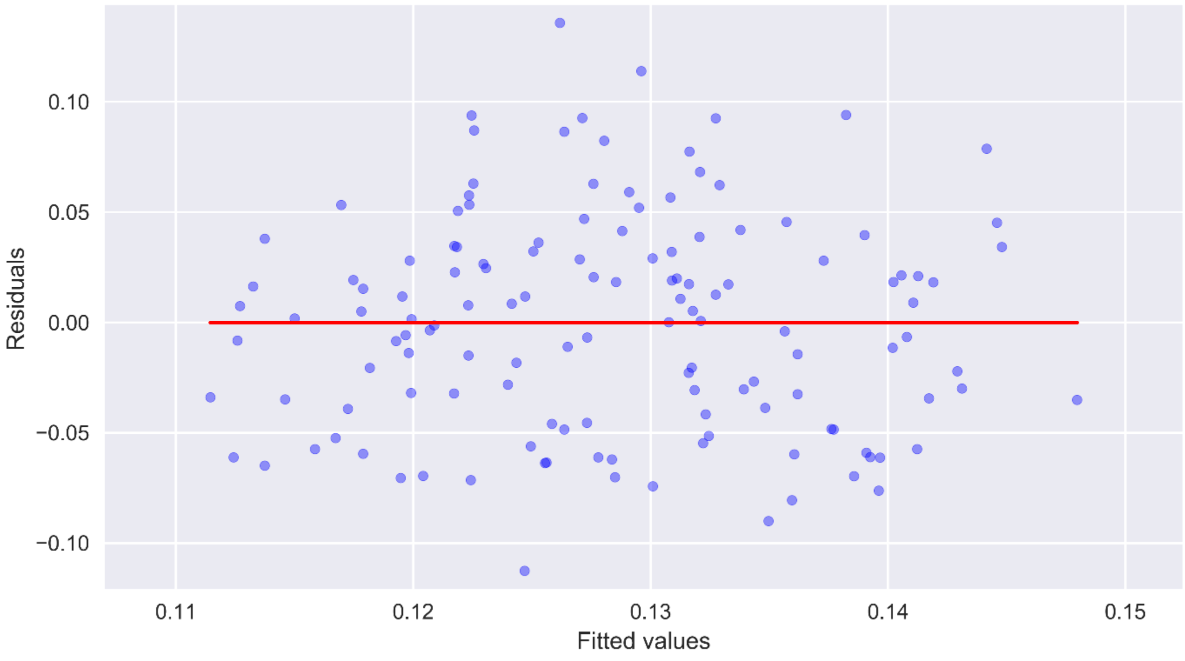

Figure A-2: Scatter plot of univariate regression residuals and fitted values from model in figure A-1.

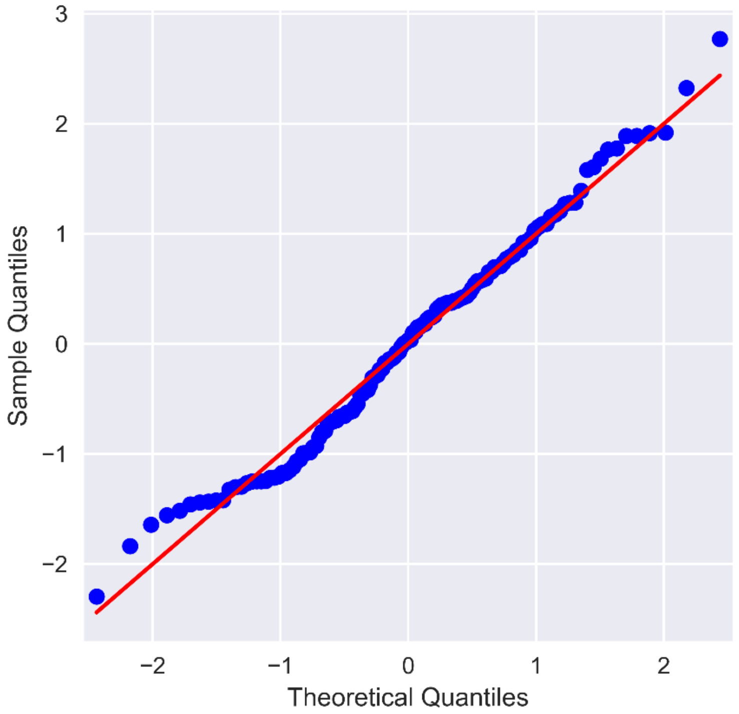

Figure A-3: QQ-plot of univariate regression model in figure A-1.

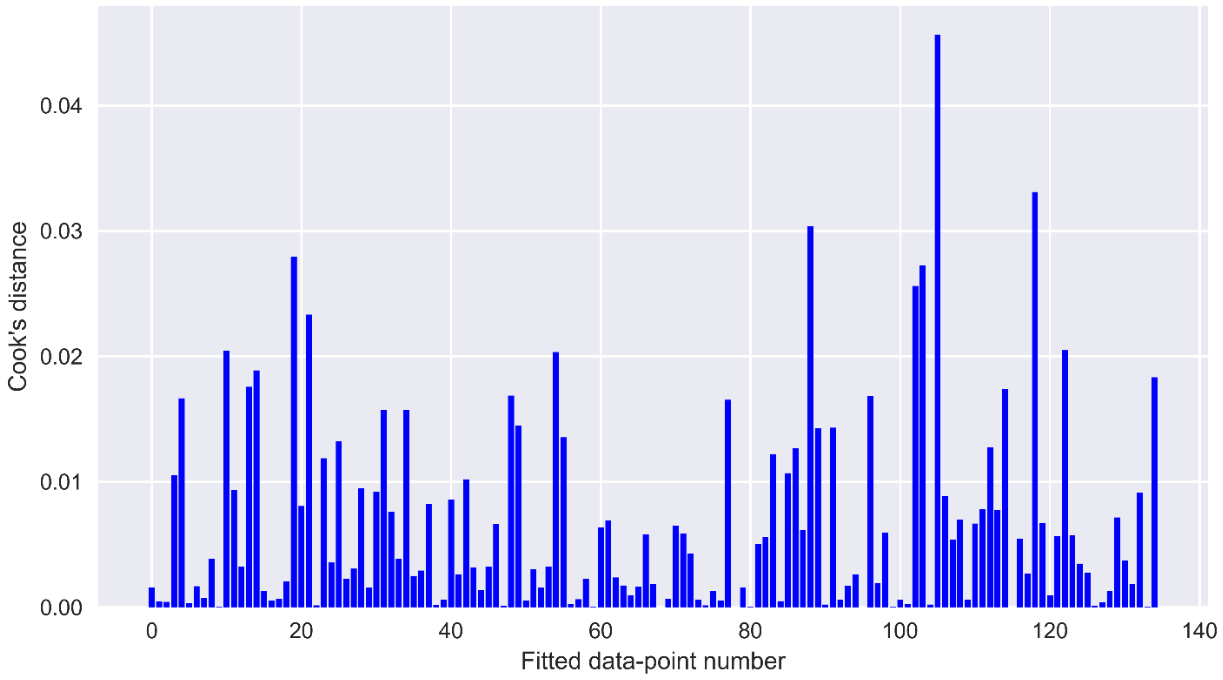

Figure A-4: Cook's distance values for each fitted data-point from model in figure A-1.
