# Supplementary material for: Exploring emergency department 4-hour target performance and cancelled elective operations: a regression analysis of routinely collected and openly reported NHS trust data
Source: BMJ Open. 2018 May 24;8(5):e020296. doi: 10.1136/bmjopen-2017-020296 (PMC5988090; doi:10.1136/bmjopen-2017-020296)
Supplement: Author's manuscript [file bmjopen-2017-020296.draft_revisions.pdf]

BMJ Open

BMJ Open is committed to open peer review. As part of this commitment we make the peer review history of every article we publish publicly available.

When an article is published we post the peer reviewers' comments and the authors' responses online. We also post the versions of the paper that were used during peer review. These are the versions that the peer review comments apply to.

The versions of the paper that follow are the versions that were submitted during the peer review process. They are not the versions of record or the final published versions. They should not be cited or distributed as the published version of this manuscript.

BMJ Open is an open access journal and the full, final, typeset and author-corrected version of record of the manuscript is available on our site with no access controls, subscription charges or pay-per-view fees (http://bmjopen.bmj.com).

If you have any questions on BMJ Open's open peer review process please email info.bmjopen@bmj.com

BMJ Open

Exploring emergency department four-hour target performance and cancelled elective operations. A regression analysis of routinely collected and openly reported NHS trust data.

Journal:	*BMJ Open*
Manuscript ID	bmjopen-2017-020296
Article Type:	Research
Date Submitted by the Author:	25-Oct-2017
Complete List of Authors:	Keogh, Brad; University of Southampton Faculty of Health Sciences<br>Culliford, David; University of Southampton, Faculty of Health Sciences<br>Guerrero-Luduena, Richard; University of Southampton Faculty of Health Sciences<br>Monks, T; University of Southampton Faculty of Health Sciences
Keywords:	4 hour target, performance target, A&E, Accident & Emergency, five year forward view

SCHOLARONE™
Manuscripts

For peer review only

Exploring emergency department four-hour target performance and cancelled elective operations. A regression analysis of routinely collected and openly reported NHS trust data.

Brad Keogh*, David Culliford, Richard Guerrero-Luduena, Thomas Monks

NIHR CLAHRC Wessex, University of Southampton, Building 67 E2005, Southampton, SO17 1BJ
Brad Keogh
Research Fellow

NIHR CLAHRC Wessex, University of Southampton, Building 67 E2005, Southampton, SO17 1BJ
David Culliford
Senior Medical Statistician

Centre for Implementation Science, University of Southampton, Building 67 E2007, Southampton, SO17 1BJ
Richard Guerrero-Luduena
Research Fellow

NIHR CLAHRC Wessex, University of Southampton, Building 67 E2005, Southampton, SO17 1BJ
Thomas Monks
Principal Research Fellow

* Correspondence to: brad.keogh@soton.ac.uk

For peer review only

ABSTRACT

Objective To quantify the effect of intra-hospital patient flow on Emergency Department (ED) performance targets and indicate if the expectations set by the NHS England five year forward review are realistic in returning emergency services to previous performance levels.

Design Linear regression analysis of routinely reported trust activity and performance data.

Setting NHS trusts in England submitting routine nationally reported measures to NHS England.

Participants 142 acute non-specialist trusts operating in England between 2012 and 2016.

Main outcome measures The primary outcome measures were: proportion of four-hour waiting time breaches and cancelled elective operations.

Methods Univariate and multivariate linear regression models were used to show relationships between each of the outcome measures, and various measures of trust activity: empty day-beds, empty night-beds, delayed transfers of care, day to night bed ratio, ED conversion ratio, ratio of emergency to elective admissions, and emergency admissions per bed ratio.

Results Univariate regression results using the outcome of four-hour breaches showed clear relationships with: empty night-beds and ED conversion ratio between 2012-2016. The day to night bed ratio showed a negative relationship and an increasing ability to explain variation in performance between 2015-2016. Delayed transfers of care showed little evidence of an association. Multivariate model results indicated that the ability of patient flow variables to explain four-hour target performance had reduced (19% to 12%) between 2012-2016.

Conclusions The flow of patients through trusts is shown to influence ED performance, however performance has become less explainable by intra-trust patient flow between 2012 and 2016. Some commonly stated explanatory factors such as delayed transfers of care showed limited evidence of being related. The results indicate some of the measures proposed by NHS England to reduce pressure on EDs may not have the desired impact on returning services to previous performance levels.

Strengths and limitations of this study

- This study is the first to examine in detail the in-hospital patient-flow factors that influence ED four-hour performance.
- We have analysed the change in the importance of common explanatory theories of hospital flow bottlenecks across five years using recent openly published data.
- There are some reports of ‘gaming’ of the four-hour ED target which are submitted to NHS England, however this is the measure which trusts are judged and funded therefore it is argued that it is suitable for use within this study.
- This study focusses on the macro-flow of patients across trusts. Future work investigating the relative importance of macro-flow, the micro-flow within ED departments and population factors would be of value to assess the different pressures trusts face.

For peer review only

INTRODUCTION

Background

It is widely reported that pressures on acute NHS trusts across England have been steadily increasing in recent years [1–5] . This is often reported in the media as rising numbers of breaches of the four-hour target, which is calculated as the percentage of patients being treated within four hours of arriving at an Emergency Department (ED). Concerns over the number of cancelled elective operations at trusts, as a result of increasing emergency pressures, have also been highlighted [5]. There is increasing pressure for NHS services to return to a 95% adherence of the four-hour target as part of the NHS England 'Next steps on the NHS five year forward view'[6], which has received some criticism [7]. Deliverables have been set which include looking to increase 'front door streaming', improving patient flow and reducing delayed transfers of care. There is currently uncertainty around the impact these interventions will have and if they are likely to result in a return to the performance targets expected.

There are several analyses which aim to understand the causes for ED performance decline and increasing pressure on acute services in England over recent years. Some analyses and commentary have suggested that high rates of bed occupancy and delayed transfers of care within trusts could be increasing the pressure on acute services, and in some cases may lead to increased waiting times in ED departments [3,8–11]. However, there is currently limited peer-reviewed statistical evidence showing the relationship between these factors and routinely collected measures of pressure on acute trusts. One of the few peer-reviewed works that has been published [12] examined data over a single two-week period in 2002 and used linear regression analysis to show a relationship between hospital bed occupancy and four-hour target performance. The study is univariate however and is limited to only a small time-window, hence it only provides a limited amount of understanding of the contributing factors to four-hour target performance. Other quantitative studies investigating the cases and consequences around waiting times in EDs [13–21] have looked at data which is not openly published and often pertains only to a small number or single hospital not necessarily located in the UK. Many of these studies also only look at the ED in isolation. These studies have limited scope in explaining the relative importance of different factors affecting growing emergency pressures on hospitals in England.

A systematic quantitative study is required which looks at the patient flow factors across trusts to understand the factors that trusts can modify which could improve performance and care. Greater understanding of the factors may allow appropriate targeting of resources tailored to trusts, rather than a suite of measures which are expected to be implemented across all providers. The aim of this work is to investigate the relative impact of the commonly highlighted variables: bed occupancy, delayed transfers of care, and other routinely measured operational factors, on the four-hour target as well as on cancelled elective operations across Acute trusts in England. This will provide evidence of the relative importance of each of these factors as well as how these dependencies have changed over time. It will also demonstrate how routinely collected and openly reported data can be utilised in a statistically robust way to understand more about how pressures on NHS services are changing.

Simplified high level system flow

Figure 1 shows a simplified hospital trust system and the patient flows through it. Patients attending ED will either be admitted as an inpatient to the trust or will be discharged from the ED. Patients may only be admitted from ED to the inpatient provision if there is space available (usually in the form of a bed). Space will only become available as inpatients are discharged or transferred from the trust to: home, social care provision or another trust. Additionally, there is pressure to admit from patients undergoing elective (non-emergency) procedures. Any of these patient flows could be a bottleneck which can result in a deterioration of trust performance measures.

Two measures of the pressure a trust is facing are breached attendances and cancelled elective operations; these are commonly thought to be a measure of pressure on EDs and inpatient provision respectively. There are several factors, related to patient flow, which may provide insight into the pressures that trusts are currently facing. Those which will be investigated in this study are related to: bed occupancy, which is reported both in day and night beds; day to night bed ratio, indicating the split between bed types in a trust; delayed transfers of care; conversion ratio, the proportion of patients attending ED who are admitted; casemix ratio, indicating the split between emergency

For peer review only

and elective admissions; and the number of admissions per bed within a trust. Figure 1 highlights these measures around the area they are likely to affect the hospital system most acutely.

Figure 1: a simplified trust system broadly illustrating patient flow.

Internal workings of an ED

The efficiency of internal ED micro-flow has been analysed extensively using modelling and simulation[22–24]. This, for example, has explored the use of fast tracks for minor injuries or patient 'streaming' (now common place in the England[25]; prioritisation by acuity[17]; and workforce scheduling and resourcing[26]. Processes of patient flow through ED and into trusts have also been shown to vary considerably between sites [17]. However, our unit of analysis is the hospital and its effects on four-hour performance.

METHODS

The study followed the STROBE RECORD reporting guidelines [27].

Data collation

A data set was collated from published open-data hosted on the NHS England Statistics website [28].These data sets included data for all NHS trusts in England, as well as minor injury units and walk-in-centres. At the time of collation data was available from quarter 2, 2011 until quarter 4, 2016 (calendar year) for the statistical reports entitled:

- Emergency department attendances and emergency admissions
- Bed availability and occupancy
- Cancelled elective operations
- Delayed transfers of care
- Hospital activity

The data source is created from each NHS organisation routinely reporting their own counts of activity, which NHS England collate and publish. This is a separate source from the Secondary Uses Service or Hospital Episodes Statistics. Full definitions of the indicators and the rules for submission by the providers are available from NHS England [28].

Trust filtering

The collated dataset was filtered to only include NHS trusts in England defined as: Small, Medium, Large Acute and Teaching. These trusts are the organisations who have seen the greatest reduction in the four-hour target. Mental health trusts, acute specialist trusts, walk in centres, practices, health centres, out-of-hours services and treatment centres were all excluded from the analysis. The definitions for NHS trust types are available online [29].

Study variables

The collated data contained counts of events for each NHS trust in England, which were converted into a proportion or a ratio using an appropriate denominator specific to the same trust for each time-period. For example: the 'number of attendances in ED lasting greater than four-hours' was divided by 'total number of ED attendances' of the period at that trust, and the 'number of beds occupied' were divided by 'total number of beds'. This created variables, which allowed useful comparisons between trusts of different sizes and activity levels. A summary of the variables investigated in this study are included in table 1, along with information on how they were calculated and a description of how they should be interpreted. The variables in this study were created from aggregating quarterly data for each year between (2012-2016) and relate to those in figure 1.

For peer review only

Table 1: Variables included in the study.

Variables	Type	Numerator	Denominator	Units	Transformation applied?	Interpretation
Breached attendances	Outcome	Number of ED attendances greater than 4 hours	Number of ED attendances	-	-	Proportion of ED attendances waiting >4hrs.
Cancelled electives	Outcome	Number of cancelled elective operations	Number of elective admissions	Operations per admission	log	Ratio of cancelled elective operations to elective admissions. In absence of number of planned elective operations this is the most suitable denominator.
Empty day beds	Explanatory	Number of unoccupied day beds	Number of day beds	-	Categorised (5)	Ratio of unoccupied day beds to total number of day beds.
Empty night beds	Explanatory	Number of unoccupied night beds	Number of night beds	-	log	Ratio of unoccupied night beds to total number of night beds.
Delayed transfers	Explanatory	Number of bed days taken by delayed transfers	Number of night beds	10 bed-days	-	The number of bed-days lost to delayed transfers for each night bed at a trust, over the course of a year.
Day to night bed ratio	Explanatory	Number of day beds	Number of night beds	-	-	Ratio of total day beds to total night beds.
ED conversion ratio	Explanatory	Number of emergency admissions via ED	Number of attendances at ED	-	-	Ratio of ED admissions to attendances. Often commonly referred to as 'Conversion ratio'.
Admission casemix ratio	Explanatory	Number of non-elective (emergency) admissions	Number of elective admissions	-	log	Proportion of admissions that are emergency or ratio of emergency to elective admissions.
Emergency admission/ bed ratio	Explanatory	Number of non-elective (emergency) admissions	Number of day and night beds	10 Admissions	-	Number of emergency admissions per bed over the course of a year.

For peer review only

Variable distributions and transformation

After conversion into proportions/ratios, non-normal variables were transformed using a natural log function (cancelled electives, empty night beds, admission casemix ratio). One variable (empty day beds) contained zero values and hence a log transformation was not appropriate. This variable was categorised. Where this was conducted bin sizes were created based on data across all years of study in order to provide consistent transformation.

Bias

Some missing data was found where trusts had not submitted data. A maximum of 4% of trusts were found to have missing data for any variable in any year. Our initial protocol intended to blind the variables throughout the analysis, however variables were un-blinded part way through the study as it was decided that greater contextual understanding of the problem was required to fully develop the analysis.

Statistical methods

Univariate ordinary least squares linear regression was conducted using breached attendances as the outcome variable against each of the explanatory variables. To ascertain how the importance of each variable has changed over time the regression analysis was performed for each year separately. As this method could present a statistical problem of multiple comparisons, undue emphasis was not placed on statistical significance tests. Results are presented as an exploratory study, showing the regression coefficients, associated confidence intervals and coefficient of determination values in each case. Only consistent associations which are of clinical importance are highlighted in the discussion. Multivariate regression was also performed to ascertain the relative importance of each outcome variable on breached attendances when combined into a single model. A model containing all outcome variables considered of interest in the univariate regression analysis was created to provide some understanding of the interaction between outcome variables. The univariate and multivariate models' residuals were checked visually for normality and homogeneity. Influential outliers with high leverage were also investigated using Cook's distance. Abnormalities are reported in the results section. The same method of analysis was repeated using cancelled elective operations as the explanatory variable.

All the analysis conducted in this work was completed with the python language (version 3.6.0, www.python.org) using the: Statsmodels (version 0.8.0, www.statsmodels.org) and Pandas (version 0.19.2, pandas.pydata.org) libraries. This was done in an Anaconda environment (www.anaconda.com) utilising Jupyter notebooks (jupyter.org).

RESULTS

In 2012 there were 254 organisations in England reporting at least one of the variables investigated in this study (226 in 2016). Figure 2 shows the distribution of four-hour target breaches by organisation type. The acute and teaching trusts are observed to have higher proportions of breaches, with increases between 2012 and 2016. Most non-acute and specialist trusts still conform to the four-hour target in 2016. Hence the former group were the focus of this study. The number of trusts reduced to 142 when applying the criteria of the organisation type for the study (135 in 2016). Missing data reduced the number of trusts for some variables to 136 (131 in 2016).

Figure 2: the distribution of four-hour target breaches by organisation type for 2012 and 2016.

How variables have changed over time

Table 2 quantifies changes of each variable between 2012-2016. Between 2012 and 2016 the median values of breached attendances and cancelled elective operations has increased. The spread of the values has also increased in the time period in both cases, however more noticeably by over three times in the case of 'breached attendances'. The most noticeable increases occurred between 2015 and 2016 in both cases. There has been a decline the

For peer review only

proportion of empty night beds between 2015 and 2016 (it should be noted that this variable is the reverse of occupancy; 'empty beds' = 1 – 'occupancy'). This reflects the historical reduction in night beds which has been noted elsewhere [10]. Between 2012 and 2016 there has been a noticeable increase in delayed transfers and emergency admission to bed ratios across trusts. Median bed-days and IQR lost due to delayed transfers has doubled over the period. Less prominent increases are observed in: empty day beds, day to night bed ratio and admission casemix ratio over this period. Although there are some fluctuations in ED conversion ratio, there is no overall change across the period.

Table 2: Changes in distribution of explanatory variables between 2012 and 2016.

Variable		2012	2013	2014	2015	2016
Breached attendances	median	0.05	0.05	0.06	0.07	0.13
	IQR	0.02	0.02	0.03	0.06	0.07
	5-95%	0.05	0.07	0.10	0.11	0.16
Cancelled electives	median	0.012	0.013	0.013	0.013	0.015
	IQR	0.007	0.008	0.010	0.010	0.011
	5-95%	0.019	0.019	0.020	0.021	0.026
Empty day beds	median	0.10	0.10	0.09	0.11	0.11
	IQR	0.17	0.17	0.17	0.17	0.21
	5-95%	0.38	0.37	0.43	0.40	0.37
Empty night beds	median	0.13	0.13	0.13	0.13	0.11
	IQR	0.08	0.08	0.09	0.08	0.07
	5-95%	0.16	0.17	0.18	0.18	0.14
Delayed transfers	median	0.73	0.77	0.91	1.04	1.36
	IQR	0.76	0.71	0.75	1.05	1.18
	5-95%	1.56	1.72	1.88	2.25	2.99
Day to night bed ratio	median	0.10	0.10	0.10	0.11	0.11
	IQR	0.07	0.07	0.07	0.08	0.08
	5-95%	0.14	0.17	0.17	0.18	0.17
ED conversion ratio	median	0.22	0.23	0.23	0.24	0.23
	IQR	0.09	0.09	0.10	0.09	0.08
	5-95%	0.19	0.19	0.19	0.19	0.19
Admission casemix ratio	median	1.15	1.13	1.16	1.17	1.19
	IQR	0.39	0.51	0.45	0.41	0.40
	5-95%	1.12	1.21	1.59	1.55	1.67
Emergency admission/ bed ratio	median	4.42	4.47	4.62	4.76	4.93
	IQR	0.94	1.10	1.05	0.99	1.08
	5-95%	2.43	2.45	2.61	2.65	2.69

Breached Attendances: Univariate regression analysis

Table 3 gives the results of the univariate regression models using breached attendances as the outcome variable. Night bed emptiness and ED conversion ratio showed respectively positive and negative associations, with breached attendances, consistently for each year of study. For night bed emptiness in 2012 the R^2 value 0.10 is of similar magnitude to a previous study [12], however in subsequent years is observed to reduced. The day to night bed ratio showed a negative relationship with trust breaches, which increased in strength during 2015-2016; it also shows an increasing ability to account for breaches (increasing R value between 2014-2016). The delayed transfers variable showed little evidence of association with breached attendances between 2012-2015.

Table 3: univariate regression for the breached attendances outcome variable. Note: all results were based on data from 131 or more trusts (* indicates non-normality, heteroskedasticity or influential outlier in regression).

For peer review only

Variable	Parameter	Year				
		2012	2013	2014	2015	2016
Empty day beds	R^2	0.02	0.01	0.01*	0.00*	0.00
	gradient	-0.001	-0.001	0.001	0.001	0.002
	(95% CIs)	(-0.003,0.001)	(-0.004,0.002)	(-0.002,0.005)	(-0.003,0.006)	(-0.004,0.008)
	p	0.15	0.43	0.4	0.54	0.6
Empty night beds	R^2	0.10*	0.09	0.07	0.05	0.06*
	gradient	-0.012	-0.015	-0.017	-0.017	-0.025
	(95% CIs)	(-0.019,-0.006)	(-0.022,-0.007)	(-0.027,-0.006)	(-0.031,-0.004)	(-0.042,-0.008)
	p	<0.01	<0.01	<0.01	0.01	<0.01
Delayed transfers	R^2	0.04*	0.02	0.01*	0.01	0.04
	gradient	0.006	0.005	0.003	0.003	0.011
	(95% CIs)	(0.001,0.011)	(-0.001,0.011)	(-0.004,0.011)	(-0.005,0.011)	(0.002,0.020)
	p	0.01	0.13	0.36	0.43	0.02
Day to night bed ratio	R^2	0.03	0.02	0.01	0.04	0.05
	gradient	-0.058	-0.066	-0.058	-0.146	-0.218
	(95% CIs)	(-0.118,0.002)	(-0.138,0.006)	(-0.156,0.040)	(-0.264,-0.027)	(-0.378,-0.058)
	p	0.06	0.07	0.25	0.02	0.01
ED conversion	R^2	0.07	0.05	0.05*	0.05	0.03
	gradient	0.071	0.082	0.114	0.143	0.144
	(95% CIs)	(0.027,0.115)	(0.025,0.140)	(0.033,0.195)	(0.039,0.248)	(0.000,0.288)
	p	<0.01	0.01	0.01	0.01	0.05
Admission casemix	R^2	0.00	0.00	0.00	0.00*	0.00
	gradient	0.002	0.001	-0.002	0.007	-0.001
	(95% CIs)	(-0.007,0.010)	(-0.010,0.012)	(-0.016,0.012)	(-0.012,0.025)	(-0.027,0.024)
	p	0.70	0.86	0.77	0.47	0.93
Emergency admission to bed ratio	R^2	0.01	0.02	0.01*	0.04	0.01
	gradient	0.002	0.003	0.003	0.009	0.005
	(95% CIs)	(-0.001,0.006)	(-0.001,0.008)	(-0.002,0.009)	(0.001,0.017)	(-0.005,0.015)
	p	0.21	0.14	0.25	0.03	0.33

Breached attendances: Multivariate regression analysis

Table 4 shows the results of the multivariate regression for 2012 and 2016. In 2012 empty night beds and ED conversion ratio variables were both statistically significant and the model was able to explain 19% of the variation in the breached attendances variable. In 2016 the results of the multivariate model show only day to night bed ratio to be statistically significant in predicting breached attendances. The R^2 value indicates that only 12% of the variation in breached attendances can be accounted for with the parameters investigated in this study in 2016. When applying the multivariate model for other years a reduction in the importance of empty night beds and ED conversion ratio, and an increase in importance of day to night bed ratio was steadily observed between 2012-2016.

Table 4: multivariate regression model output for breached attendances as explanatory variable (R^2 = 0.19 in 2012, R^2 = 0.1 in 2016).

	2012			2016		
Variable	Gradient	p	95% CIs	Gradient	p	95% CIs
Empty night beds	-0.010	<0.01	-0.016 -0.004	-0.016	0.09	-0.034 0.003
Delayed transfers	0.004	0.12	-0.001 0.008	0.006	0.24	-0.004 0.015
Day to night bed ratio	-0.045	0.12	-0.102 0.012	-0.169	0.04	-0.330 -0.008

For peer review only

ED conversion ratio	0.068	<0.01	0.025<br>0.112	0.104	0.17	-0.044<br>0.252

Cancelled elective operations: Univariate regression analysis

Table 5 gives the results of the univariate regression models using cancelled elective operations as the outcome variable. ED conversion ratio, admission casemix ratio and delayed transfers showed a positive relationship with cancelled elective operations. Between 2014-2016 the variables ED conversion ratio and admission casemix ratio show increasingly positive associations with cancelled elective operations. These variables also demonstrate a relatively high ability to explain the outcome variable in comparison to the other variables in the study (R^2 values 0.07 & 0.11 respectively in 2016). The delayed transfers variable is observed to increase in importance between 2013-2016. The other variables included in the study did not show evidence of any clear association with cancelled elective operations.

Table 5: univariate regression output for the cancelled elective operations outcome variable (* indicates non-normality, heteroskedasticity or influential outlier in regression).

Variable	Parameters	Year				
		2012	2013	2014	2015	2016
Empty day beds	R^2	0.00	0.00	0.00	0.00	0.00
	gradient	0.009	0.009	0.008	-0.014	-0.003
	(95% CIs)	(-0.047,0.066)	(-0.046,0.064)	(-0.051,0.066)	(-0.079,0.051)	(-0.069,0.062)
	p	0.75	0.75	0.8	0.68	0.92
Empty night beds	R^2	0.01	0.02	0.00	0.00	0.02
	gradient	-0.135	-0.130	-0.087	-0.066	-0.149
	(95% CIs)	(-0.328,0.059)	(-0.307,0.047)	(-0.273,0.099)	(-0.260,0.129)	(-0.336,0.039)
	p	0.17	0.15	0.36	0.51	0.12
Delayed transfers	R^2	0.01	0.00*	0.01*	0.03*	0.03
	gradient	0.087	0.040	0.065	0.108	0.103
	(95% CIs)	(-0.064,0.237)	(-0.097,0.178)	(-0.063,0.192)	(-0.007,0.222)	(0.004,0.201)
	p	0.26	0.56	0.32	0.07	0.04
Day to night bed ratio	R^2	0.01	0.00	0.00	0.00	0.00
	gradient	1.067	0.589	0.245	0.255	-0.677
	(95% CIs)	(-0.733,2.868)	(-0.997,2.175)	(-1.410,1.900)	(-1.444,1.954)	(-2.437,1.082)
	p	0.24	0.46	0.77	0.77	0.45
ED conversion	R^2	0.05	0.02	0.07	0.06	0.07
	gradient	1.728	1.020	2.275	2.108	2.419
	(95% CIs)	(0.402,3.055)	(-0.288,2.327)	(0.914,3.637)	(0.627,3.589)	(0.901,3.937)
	p	0.01	0.13	<0.01	<0.01	<0.01
Admission casemix	R^2	0.02	0.05	0.13	0.12*	0.11
	gradient	0.240	0.318	0.507	0.524	0.518
	(95% CIs)	(-0.016,0.496)	(0.082,0.553)	(0.288,0.726)	(0.275,0.772)	(0.258,0.778)
	p	0.07	<0.01	<0.001	<0.001	<0.001
Emergency admission to bed ratio	R^2	0.00	0.00	0.00	0.00	0.00
	gradient	0.029	0.010	0.009	-0.025	-0.033
	(95% CIs)	(-0.077,0.136)	(-0.087,0.108)	(-0.089,0.107)	(-0.136,0.087)	(-0.143,0.077)
	p	0.58	0.84	0.85	0.66	0.56

Cancelled electives: Multivariate regression analysis

Table 6 shows a summary of the variables included in the multivariate linear regression model using cancelled elective operations as an explanatory variable in 2012 and 2016. In 2012 the only statistically significant variable

For peer review only

For peer review only - http://bmjopen.bmj.com/site/about/guidelines.xhtml

related to cancelled elective operations was ED conversion ratio, which had a positive association. In 2016 the results indicate that the statistically significant variables in explaining variation were ED conversion ratio and admission casemix ratio. Both of these variables were observed to be statistically significant in the model for year between 2014-2016. Overall the model was able to account for 17% of the variation in cancelled elective operations in 2016, which demonstrates an increasing ability to determine cancelled elective operations over the study period (increasing from 7% in 2012).

Table 6: multivariate regression model output for cancelled electives as explanatory variable (R^2 = 0.07 in 2012; R^2 = 0.17 in 2016).

	2012			2016		
Variable	Gradient	p	95% CIs	Gradient	p	95% CIs
Delayed transfers	0.067	0.37	-0.081 0.215	0.070	0.15	-0.025 0.164
ED conversion ratio	1.625	0.02	0.302 2.949	1.881	0.01	0.385 3.377
Admission casemix ratio	0.225	0.08	-0.027 0.477	0.483	<0.001	0.230 0.736

DISCUSSION

Summary of findings

The intra-hospital patient flow variables with greatest association with better acute trust four-hour target performance in England were found to have varied over the period 2012-2016. The main variables of interest in explaining performance in 2012 were found to be the proportion of empty night beds and ED conversion ratio. These variables were seen to have reducing importance over the subsequent years in the multivariate model. The day to night bed ratio variable was observed to have increased in importance between 2012 and 2016, and in the years 2015-2016 it was the most important patient flow factor in explaining four-hour target performance in the multivariate model. Multivariate model results also show that intra-hospital patient flow is only responsible for explaining some of the variation in four-hour target performance, and between 2012-2016 demonstrated a reducing ability to explain this variation (R^2 value of model reducing from 0.19 to 0.12). There was limited evidence of a clear association between delayed transfers of care and four-hour target performance, either in the univariate or multivariate model results.

The intra-hospital patient flow variables associated with higher levels of cancelled elective operations at acute trusts between 2012-2016 were ED conversion ratio and the admission casemix ratio. Between 2012-2016 the importance of the admission casemix ratio is observed to have increased beyond that of ED conversion ratio. The ability of the multivariate model to explain variation in cancelled elective operations has increased between 2012 and 2016 (R^2 value of model increasing from 0.07 to 0.17), indicating that the emergency workload that trusts face are becoming an increasingly important factor in elective procedures being cancelled in England.

How important is intra-hospital flow to the four-hour target?

The ability of the multivariate model to explain only 12% of the variation in four-hour target in 2016 (which has reduced since 2012 from 17%) indicates that there are other factors that are increasingly important in determining four-hour performance. There are a number of factors which may affect ED processes and performance measures: macro or intra-hospital flow (i.e. patient flows across the whole trust, such as those investigated in this study); micro-flow factors within departments/wards (i.e. staffing[4,17], work flows[25,30], access to diagnostics[17]); population factors (i.e. age, sex, deprivation[31], access to GP/community/social care services[32]); noise (i.e. recording errors, reporting differences[10], 'gaming' of four-hour target[5,17]). If the mechanisms of pressures facing acute trusts are to be understood more fully, future work is required to quantify the relative effects of each of these factors on trust performance measures.

For peer review only - http://bmjopen.bmj.com/site/about/guidelines.xhtml

Bed occupancy

In our univariate analysis night-bed occupancy was consistently associated with 4-hr performance (2012-2016). This is a result consistent with queuing theory. That is, if trusts do not provide adequate bed 'buffer' capacity to cope with the peaks of emergency admission demand then ED performance will decline. As such trusts must continue to focus on innovations to reduce night-bed occupancy. Targets such as 85% occupancy have been proposed elsewhere [33] although this seems unrealistic given in 2012 and 2016 respectively only 39% and 25% of trusts attained this level of night-bed occupancy (or lower).

Day to night bed ratio and using day beds as 'buffers'

Our results show that that trusts with higher ratio of day to night bed capacity were more likely to have higher four-hour performance in 2016. It has been highlighted that admission and discharge patterns through trusts have peaks at different times of day [9,11]. Hence an explanation for this result is that day-bed capacity can be used flexibly as a temporary 'buffer' for patient admission whilst the discharge of patients from the trust catches up during the day. Day beds are defined as consultant-led beds that are closed overnight [28], hence patients are not able to occupy them for long periods as in the case of night beds, and the occupancy of such wards/areas will be low at the beginning of each day to allow admissions. It is possible that more trusts are taking advantage of this flexibility as the day to night bed ratio of trusts in England has increased between 2012-2016 (see table 2). This may be in a response to trusts operating at greater levels of occupancy (also see table 2) towards the end of this period, and requiring a way of ensuring bed 'buffers' are available to allow patients to be admitted more promptly. We note that if occupancy can be reduced this may remove the need to use day beds as internal buffers.

Delayed transfers

Our expectations were that delayed transfers of care would be a strong predictor of four-hour performance. Our results do not support this commonly held assumption. This suggests that hospital initiatives to reduce delayed transfers of care may not yield the expected benefits for ED waiting times. We in no way suggest that initiatives to reduce delayed transfers are not worthwhile, as expedited transfers to appropriate care such as rehabilitation or social care have clear clinical and quality of life benefits for patients. We do note the strong association between in-patient bed occupancy (night-bed occupancy) and delayed transfers of care (Spearman's rank correlation in 2016 = -0.27, p = 0.002). However, it is clear that a focus on only delayed transfers will not reduce occupancy to a level that is sufficient to release the pressure on EDs; more holistic approaches to reducing bed occupancy may be required.

'Clinical streaming' and conversion ratio

'Clinical streaming' in ED aims to triage patients within 15minutes of their arrival, and refer patients to other appropriate services [34]. This aims to: reduce the load on EDs by only treating patients who cannot be treated by other services, and provide better patient flow within EDs by prioritising different routes of care suited to patient needs. Earlier review by senior clinicians has been shown to reduce waiting time for patients who are not admitted[30]; it is also thought to reduce avoidable admissions[35]. Hence another plausible result of clinical streaming may be a reduction in conversion ratio. Conversion ratios of trusts in England have not changed noticeably between 2012 -2016 (see table 2) whilst some trusts are already known to have introduced clinical streaming [17]. As well as it being currently unclear if there could be significant changes to trust conversion ratios with the proposed roll-out of clinical streaming by NHS England [6], the realistic benefits to ED four-hour waiting times may also be minimal based on the results of our study. Our study does indicate however that lower conversion ratio is associated with a fewer cancelled elective operations, which would be beneficial for patient care. It is also not possible to comment on the improvements clinical streaming systems may have on patient experience in EDs from our study.

Strengths and weaknesses of study

This study is the first to examine in detail the in-hospital factors that influence ED four-hour performance. We have analysed the change in the importance of common explanatory theories of hospital flow bottlenecks across five years using recent openly published data.

For peer review only

Trust four-hour target reporting is known to be affected by 'gaming' [5], which may introduce extra variability into the relationships under investigation. However, this is the measure which trusts are judged and funded, therefore it is argued that it is suitable for use within this study. The quality of data recording by trusts of the 'delayed transfers' variable has been reported to be questionable [10] which may explain the uncertainly in the importance of this variable over time.

This study focusses on the macro-flow factors across trusts. Future work including the relative importance of the macro flow, micro flow, population factors and noise would be of value to assess the different pressures trusts face and aid in the targeting of service re-configurations.

FOOTNOTES

We thank the NHS England Statistics Unify2 team, for help in accessing and understanding the open data utilised in this study.

Transparency: The lead author (the manuscript's guarantor) affirms that the manuscript is an honest, accurate, and transparent account of the study being reported; that no important aspects of the study have been omitted; and that any discrepancies from the study as planned (and, if relevant, registered) have been explained. The authors attest that we have obtained appropriate permissions and paid any required fees for use of copyright protected materials.

Datasharing: All code required to re-produce this work is available as supplementary files to the publication. The raw collated quarterly count data as well as the python code to produce the year-aggregated variable data used in this study has also been made available [36] in the hope that other researchers will be easily able to reproduce this analysis, and allow further analysis to be conducted to test other research hypotheses.

Ethics: This study was reviewed by University of Southampton ERGO ethics committee (reference 25957).

Contributors: RG created the initial research question. BK contributed to the initial design of the work and was responsible for the protocol/ethics, data acquisition and collation, coding, analysis and interpretation of results. DC was responsible for the statistical methodology and interpretation of results. TM was involved throughout the study with guidance in the design, methodology and interpretation of results. All authors were involved in the development and review of the manuscript.

Competing interests: All authors have completed the ICMJE uniform disclosure form at www.icmje.org/coi_disclosure.pdf and declare: no support from any organisation for the submitted work; no financial relationships with any organisations that might have an interest in the submitted work in the previous three years; no other relationships or activities that could appear to have influenced the submitted work.

Funding: This article presents independent research funded by the National Institute for Health Research (NIHR) Collaboration for Leadership in Applied Health Research and Care (CLAHRC) Wessex. The views expressed in this publication are those of the author(s) and not necessarily those of the National Health Service, the NIHR, or the Department of Health.

Copyright: The Corresponding Author has the right to grant on behalf of all authors and does grant on behalf of all authors, an exclusive licence (or non exclusive for government employees) on a worldwide basis to the BMJ Publishing Group Ltd to permit this article (if accepted) to be published in BMJ editions and any other BMJPGL products and sublicences such use and exploit all subsidiary rights, as set out in our licence.

REFERENCES

1. Blunt I, Edwards N, Merry L. What's behind the A&E "Crisis"? The Nuffield Trust. 2015.

For peer review only

2. Appleby J. Nuffield Winter Insight Briefing 1: Winter beds pressures. The Nuffield Trust. 2016.

3. Fisher E, Dorning H. Winter pressures: what's going on behind the scenes ? The Nuffield Trust. 2016.

4. House of Commons Health Committee. Urgent and emergency services. 2013.

5. House of Commons Health Comittee. Winter pressure in accident and emergency departments. 2016.

6. NHS England. Next Steps on the NHS Five Year Forward View. 2017.

7. Oliver D. Reducing acute admissions? a timely reality check. BMJ. 2017;357:j2082.

8. Monitor. A&E delays : Why did patients wait longer last winter? 2015.

9. Karakusevic S. Understanding patient flow in hospitals. The Nuffield Trust. 2016.

10. British Medical Association. State of the health system Beds in the NHS: England. 2017.

11. Allder S, Silvester K, Walley P. Understanding the current state of patient flow in a hospital. Clin Med (Lond). 2010;10(5):441–4.

12. Cooke MW, Wilson S, Halsall J, Roalfe A. Total time in English accident and emergency departments is related to bed occupancy. Emerg Med J. 2004;21(5):575–6.

13. Mustafa F, Gilligan P, Obu D, O'Kelly P, O'Hea E, Lloyd C, et al. "Delayed discharges and boarders": a 2-year study of the relationship between patients experiencing delayed discharges from an acute hospital and boarding of admitted patients in a crowded ED. Emerg Med J. 2016;33:636–40.

14. Sun BC, Laurie A, Prewitt L, Fu R, Chang AM, Augustine J, et al. Risk-Adjusted Variation of Publicly Reported Emergency Department Timeliness Measures. Ann Emerg Med. 2016;67(4):509–516.e7.

15. Rathlev NK, Chessare J, Olshaker J, Obendorfer D, Mehta SD, Rothenhaus T, et al. Time Series Analysis of Variables Associated With Daily Mean Emergency Department Length of Stay. Ann Emerg Med. 2007;49(3):265–71.

16. Silvester K, Harriman P, Walley P, Burley G. Does process flow make a difference to mortality and cost? An observational study. Int J Health Care Qual Assur. 2014;27(7):616–32.

17. Pinkney J, Rance S, Benger J, Brant H, Joel-Edgar S, Swancutt D, et al. How can frontline expertise and new models of care best contribute to safely reducing avoidable acute admissions? A mixed-methods study of four acute hospitals. Health Services and Delivery Research. 2016;4(3).

18. Blom MC, Jonsson F, Landin-Olsson M, Ivarsson K. Associations between in-hospital bed occupancy and unplanned 72-h revisits to the emergency department: a register study. Int J Emerg Med. 2014;7:25.

19. Graham J, Aitken ME, Shirm S. Correlation of measures of patient acuity with measures of crowding in a pediatric emergency department. Pediatr Emerg Care. 2011;27(8):706–9.

20. Harris A, Sharma A. Access block and overcrowding in emergency departments: an empirical analysis. Emerg Med J. 2010;27(7):508–11.

21. Vermeulen MJ, Ray JG, Bell C, Cayen B, Stukel TA, Schull MJ. Disequilibrium Between Admitted and Discharged Hospitalized Patients Affects Emergency Department Length of Stay. Ann Emerg Med. 2009;54(6):794–804.

22. Mohiuddin S, Busby J, Savovi J, Richards A, Northstone K, Hollingworth W, et al. Patient flow within UK emergency departments: a systematic review of the use of computer simulation modelling methods. BMJ Open. 2017;7:e015007.

23. Saghafian S, Austin G, Traub SJ. Operations research/management contributions to emergency department patient flow optimization: Review and research prospects. IIE Trans Healthc Syst Eng. 2015;5:101–23.

24. Gul M, Guneri AF. A comprehensive review of emergency department simulation applications for normal and disaster conditions. Comput Ind Eng. 2015;83:327–44.

For peer review only

25. Saghafian S, Hopp WJ, Oyen MP Van, Desmond JS, Kronick SL. Patient Streaming as a Mechanism for Improving Responsiveness in Emergency Departments. Oper Res. 2012;60(5):1080–97.

26. Duguay C, Chetouane F. Modeling and Improving Emergency Department Systems using Discrete Event Simulation. Simulation. 2007;83(4):311–20.

27. Equator network. The REporting of studies Conducted using Observational Routinely-collected health Data (RECORD) Statement.

28. NHS England. NHS England Statistics [Internet]. 2017 [cited 2017 May 1]. Available from: https://www.england.nhs.uk/statistics/

29. NHS Improvement. NHS trust list [Internet]. 2017 [cited 2017 May 1]. Available from: http://www.nrls.npsa.nhs.uk/EasySiteWeb/GatewayLink.aspx?alId=135142

30. Han JH, France DJ, Levin SR, Jones ID, Storrow AB, Aronsky D. The effect of physician triage on emergency department length of stay. J Emerg Med. 2010;39(2):227–33.

31. O'Cathain A, Knowles E, Maheswaran R, Pearson T, Turner J, Hirst E, et al. A system-wide approach to explaining variation in potentially avoidable emergency admissions: national ecological study. BMJ Qual Saf. 2014;23:47–55.

32. Flores-Mateo G, Violan-Fors C, Carrillo-Santisteve P, Peiro S, Argimon J-M. Effectiveness of organizational interventions to reduce emergency department utilization: A systematic review. PLoS One. 2012;7(5):1–7.

33. Bagust A, Place M, Posnett JW. Dynamics of bed use in accommodating emergency admissions: stochastic simulation model. BMJ. 1999;319:155–8.

34. NHS England. Clinical Streaming in the Accident and Emergency (A & E) Department [Internet]. 2017. Available from: https://www.england.nhs.uk/wp-content/uploads/2017/07/principles-for-clinical-streaming-ae-department.pdf

35. O'Cathain A, Knowles E, Turner J, Maheswaran R, Goodacre S, Hirst E, et al. Explaining variation in emergency admissions: a mixed-methods study of emergency and urgent care systems. Health Services and Delivery Research. 2014;2(48).

36. Keogh B, Culliford D, Guerrero-Luduena R, Monks T. NHS England routinely reported measures 2011-2016 [Internet]. University of Southampton [Dataset]. 2017. Available from: doi:10.5258/SOTON/D0218

For peer review only

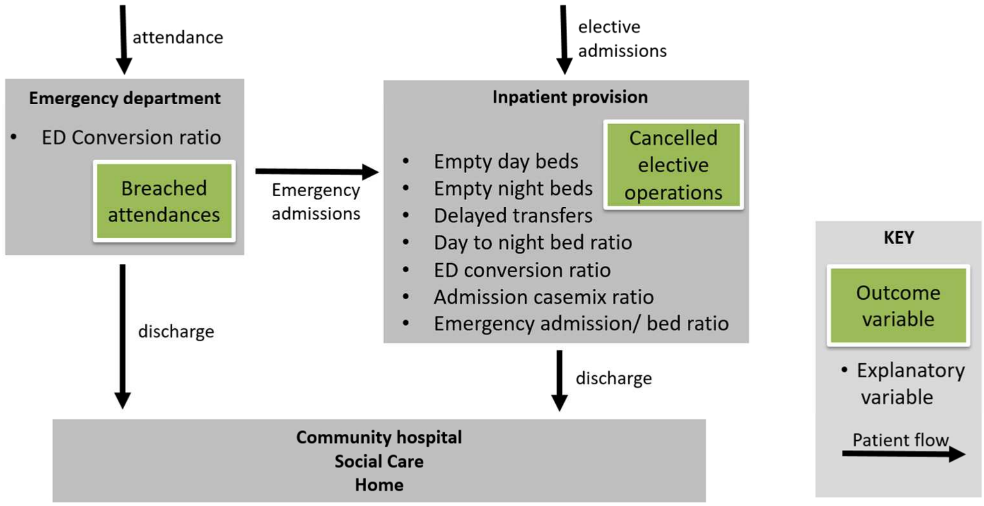

Figure 1 - a simplified trust system broadly illustrating patient flow

For peer review only - http://bmjopen.bmj.com/site/about/guidelines.xhtml

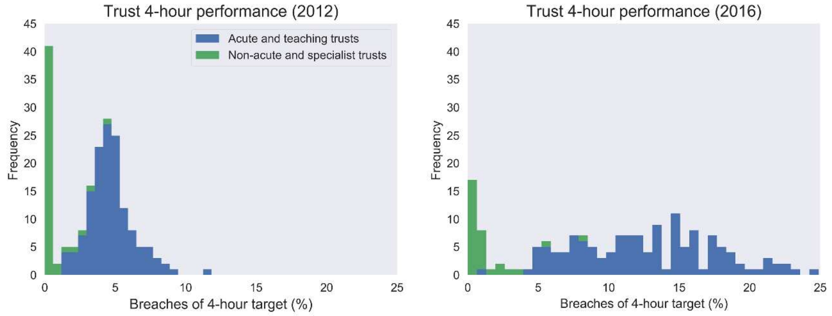

Figure 2 - the distribution of four-hour target breaches by organisation type for 2012 and 2016

For peer review only

The RECORD statement – checklist of items, extended from the STROBE statement, that should be reported in observational studies using routinely collected health data.

Author note: this study is not a traditional observational study following patients (rather it follows NHS trusts), however the RECORD statement seemed most appropriate and helpful in reporting the study. As a result some of the RECORD statements were not applicable to this study. Most of the STROBE and RECORD items are located under the corresponding section titles in the manuscript but line/page numbers have been included below (all line numbers refer to authors' line numbering).

	Item No.	STROBE items	Location in manuscript where items are reported	RECORD items	Location in manuscript where items are reported
**Title and abstract**					
	1	(a) Indicate the study's design with a commonly used term in the title or the abstract (b) Provide in the abstract an informative and balanced summary of what was done and what was found	Pages 1 & 2	RECORD 1.1: The type of data used should be specified in the title or abstract. When possible, the name of the databases used should be included.<br><br>RECORD 1.2: If applicable, the geographic region and timeframe within which the study took place should be reported in the title or abstract.<br><br>RECORD 1.3: If linkage between databases was conducted for the study, this should be clearly stated in the title or abstract.	Page 2
**Introduction**					
Background rationale	2	Explain the scientific background and rationale for the investigation being reported	Page 3		
Objectives	3	State specific objectives, including any prespecified hypotheses	line 87		
**Methods**					
Study Design	4	Present key elements of study design early in the paper	See methods section starting line 121		

For peer review only

Setting	5	Describe the setting, locations, and relevant dates, including periods of recruitment, exposure, follow-up, and data collection	Line 123		
Participants	6	*(a) Cohort study* - Give the eligibility criteria, and the sources and methods of selection of participants. Describe methods of follow-up<br>*Case-control study* - Give the eligibility criteria, and the sources and methods of case ascertainment and control selection. Give the rationale for the choice of cases and controls<br>*Cross-sectional study* - Give the eligibility criteria, and the sources and methods of selection of participants<br><br>*(b) Cohort study* - For matched studies, give matching criteria and number of exposed and unexposed<br>*Case-control study* - For matched studies, give matching criteria and the number of controls per case	Line 137	RECORD 6.1: The methods of study population selection (such as codes or algorithms used to identify subjects) should be listed in detail. If this is not possible, an explanation should be provided.<br><br>RECORD 6.2: Any validation studies of the codes or algorithms used to select the population should be referenced. If validation was conducted for this study and not published elsewhere, detailed methods and results should be provided.<br><br>RECORD 6.3: If the study involved linkage of databases, consider use of a flow diagram or other graphical display to demonstrate the data linkage process, including the number of individuals with linked data at each stage.	6.1 included line 137.<br><br>6.2 & 6.3 not relevant for this study,
Variables	7	Clearly define all outcomes, exposures, predictors, potential confounders, and effect modifiers. Give diagnostic criteria, if applicable.	Table 1; page 5	RECORD 7.1: A complete list of codes and algorithms used to classify exposures, outcomes, confounders, and effect modifiers should be provided. If these cannot be reported, an explanation should be provided.	N/A for this study
Data sources/ measurement	8	For each variable of interest, give sources of data and details of methods of assessment (measurement).	Line 123		

For peer review only

		Describe comparability of assessment methods if there is more than one group			
Bias	9	Describe any efforts to address potential sources of bias	Line 7 (following table 1)		
Study size	10	Explain how the study size was arrived at	Line 30 (following table 1)		
Quantitative variables	11	Explain how quantitative variables were handled in the analyses. If applicable, describe which groupings were chosen, and why	Line 1 (following table 1)		
Statistical methods	12	(a) Describe all statistical methods, including those used to control for confounding<br>(b) Describe any methods used to examine subgroups and interactions<br>(c) Explain how missing data were addressed<br>(d) *Cohort study* - If applicable, explain how loss to follow-up was addressed<br>*Case-control study* - If applicable, explain how matching of cases and controls was addressed<br>*Cross-sectional study* - If applicable, describe analytical methods taking account of sampling strategy<br>(e) Describe any sensitivity analyses	Line 12 (following table 1)		
Data access and cleaning methods		..		RECORD 12.1: Authors should describe the extent to which the investigators had access to the database population used to create the study population.	Line 123<br><br>Line 1 (following

For peer review only

				RECORD 12.2: Authors should provide information on the data cleaning methods used in the study.	table 1)
Linkage		..		RECORD 12.3: State whether the study included person-level, institutional-level, or other data linkage across two or more databases. The methods of linkage and methods of linkage quality evaluation should be provided.	N/A for this study
**Results**					
Participants	13	(a) Report the numbers of individuals at each stage of the study (*e.g.*, numbers potentially eligible, examined for eligibility, confirmed eligible, included in the study, completing follow-up, and analysed)<br>(b) Give reasons for non-participation at each stage.<br>(c) Consider use of a flow diagram	Line 31 (following table 1)	RECORD 13.1: Describe in detail the selection of the persons included in the study (*i.e.,* study population selection) including filtering based on data quality, data availability and linkage. The selection of included persons can be described in the text and/or by means of the study flow diagram.	Line 31 (following table 1)
Descriptive data	14	(a) Give characteristics of study participants (*e.g.*, demographic, clinical, social) and information on exposures and potential confounders<br>(b) Indicate the number of participants with missing data for each variable of interest<br>(c) *Cohort study* - summarise follow-up time (*e.g.*, average and total amount)	N/A for this study, however descriptive data from line 40 (following table 1)		
Outcome data	15	*Cohort study* - Report numbers of outcome events or summary measures over time<br>*Case-control study* - Report numbers in each exposure	N/A for this study.		

For peer review only

		category, or summary measures of exposure<br>*Cross-sectional study* - Report numbers of outcome events or summary measures			
Main results	16	(a) Give unadjusted estimates and, if applicable, confounder-adjusted estimates and their precision (e.g., 95% confidence interval). Make clear which confounders were adjusted for and why they were included<br>(b) Report category boundaries when continuous variables were categorized<br>(c) If relevant, consider translating estimates of relative risk into absolute risk for a meaningful time period	Line 54-98 (following table 1)		
Other analyses	17	Report other analyses done—e.g., analyses of subgroups and interactions, and sensitivity analyses	N/A for this study.		
**Discussion**					
Key results	18	Summarise key results with reference to study objectives	Line 101, (following table 1)		
Limitations	19	Discuss limitations of the study, taking into account sources of potential bias or imprecision. Discuss both direction and magnitude of any potential bias	Line 170 (following table 1)	RECORD 19.1: Discuss the implications of using data that were not created or collected to answer the specific research question(s). Include discussion of misclassification bias, unmeasured confounding, missing data, and changing eligibility over time, as they pertain to the study being reported.	Line 170 (following table 1)
Interpretation	20	Give a cautious overall interpretation of results considering objectives, limitations, multiplicity of	Line 119-169 (following table 1)		

For peer review only

		analyses, results from similar studies, and other relevant evidence			
Generalisability	21	Discuss the generalisability (external validity) of the study results	Discussed throughout discussion section line 101-189 (following table 1)		
**Other Information**					
Funding	22	Give the source of funding and the role of the funders for the present study and, if applicable, for the original study on which the present article is based	Line 205 (following table 1)		
Accessibility of protocol, raw data, and programming code		..		RECORD 22.1: Authors should provide information on how to access any supplemental information such as the study protocol, raw data, or programming code.	Line 191 (following table 1)

*Reference: Benchimol EI, Smeeth L, Guttmann A, Harron K, Moher D, Petersen I, Sørensen HT, von Elm E, Langan SM, the RECORD Working Committee. The REporting of studies Conducted using Observational Routinely-collected health Data (RECORD) Statement. *PLoS Medicine* 2015; in press.

*Checklist is protected under Creative Commons Attribution (CC BY) license.

For peer review only

BMJ Open

Exploring emergency department four-hour target performance and cancelled elective operations. A regression analysis of routinely collected and openly reported NHS trust data.

Journal:	*BMJ Open*
Manuscript ID	bmjopen-2017-020296.R1
Article Type:	Research
Date Submitted by the Author:	16-Feb-2018
Complete List of Authors:	Keogh, Brad; University of Southampton Faculty of Health Sciences<br>Culliford, David; University of Southampton, Faculty of Health Sciences<br>Guerrero-Luduena, Richard; University of Southampton Faculty of Health Sciences<br>Monks, T; University of Southampton Faculty of Health Sciences
**Primary Subject Heading**:	Emergency medicine
Secondary Subject Heading:	Emergency medicine
Keywords:	4 hour target, performance target, A&E, Accident & Emergency, five year forward view

SCHOLARONE™
Manuscripts

For peer review only

Exploring emergency department four-hour target performance and cancelled elective operations. A regression analysis of routinely collected and openly reported NHS trust data.

Brad Keogh*, David Culliford, Richard Guerrero-Luduena, Thomas Monks

NIHR CLAHRC Wessex, University of Southampton, Building 67 E2005, Southampton, SO17 1BJ
Brad Keogh
Research Fellow

NIHR CLAHRC Wessex, University of Southampton, Building 67 E2005, Southampton, SO17 1BJ
David Culliford
Senior Medical Statistician

Centre for Implementation Science, University of Southampton, Building 67 E2007, Southampton, SO17 1BJ
Richard Guerrero-Luduena
Research Fellow

NIHR CLAHRC Wessex, University of Southampton, Building 67 E2005, Southampton, SO17 1BJ
Thomas Monks
Principal Research Fellow

* Correspondence to: brad.keogh@soton.ac.uk

For peer review only

What is already known on this topic

Pressure on emergency departments in England has increased steadily in recent years and adherence to the four-hour performance target has reduced. There is a lack of robust information around the causes of this decline. Calls from NHS England for trusts to return to previous performance levels are accompanied with suggestions of changes to services for which the impact is unclear and unevidenced.

What this study adds

Measures affecting patient flow throughout hospital systems including: admission pressures, hospital occupancy and delayed transfers of care were collated and investigated in England between 2012-2016. The relationships between these factors, four-hour target and cancelled elective operations were quantified. The results indicate some of the measures proposed by NHS England to reduce pressure on EDs may not have the desired impact on returning services to previous performance levels.

ABSTRACT

Objective To quantify the effect of intra-hospital patient flow on Emergency Department (ED) performance targets and indicate if the expectations set by the NHS England five year forward review are realistic in returning emergency services to previous performance levels.

Design Linear regression analysis of routinely reported trust activity and performance data using a series of cross-sectional studies.

Setting NHS trusts in England submitting routine nationally reported measures to NHS England.

Participants 142 acute non-specialist trusts operating in England between 2012 and 2016.

Main outcome measures The primary outcome measures were: proportion of four-hour waiting time breaches and cancelled elective operations.

Methods Univariate and multivariate linear regression models were used to show relationships between the outcome measures, and various measures of trust activity including: empty day-beds, empty night-beds, day-to-night bed ratio, ED conversion ratio and delayed transfers of care.

Results Univariate regression results using the outcome of four-hour breaches showed clear relationships with: empty night-beds and ED conversion ratio between 2012-2016. The day-to-night bed ratio showed an increasing ability to explain variation in performance between 2015-2016. Delayed transfers of care showed little evidence of an association. Multivariate model results indicated that the ability of patient flow variables to explain four-hour target performance had reduced between 2012-2016 (19% to 12%), and had increased in explaining cancelled elective operations (7% to 17%).

Conclusions The flow of patients through trusts is shown to influence ED performance, however performance has become less explainable by intra-trust patient flow between 2012 and 2016. Some commonly stated explanatory factors such as delayed transfers of care showed limited evidence of being related. The results indicate some of the measures proposed by NHS England to reduce pressure on EDs may not have the desired impact on returning services to previous performance levels.

For peer review only

For peer review only - http://bmjopen.bmj.com/site/about/guidelines.xhtml

Strengths and limitations of this study

- This study is the first to examine in detail the in-hospital factors that influence ED four-hour performance.
- We analyse the change in the importance of common explanatory theories of hospital flow bottlenecks across five years using recent openly published data.
- There are potentially some data quality issues around the variables used in the analysis which may influence the conclusions.
- Although a relatively simple methodology was used to ensure transparency of the study, it is possible that some statistical inference from the data is lost which more complex methodologies might reveal.
- Future work involving the use of more complex statistical methodologies and the investigation of the relative importance of: patient flows within trusts, population factors and data quality, would be of use to further understand the different pressures trusts face and aid in the targeting of service re-configurations.

For peer review only

For peer review only - http://bmjopen.bmj.com/site/about/guidelines.xhtml

INTRODUCTION

Background

It is widely reported that pressures on acute NHS trusts across England have been steadily increasing in recent years [1–5] . This is often reported in the media as rising numbers of breaches of the four-hour target, which is calculated as the percentage of patients being treated within four hours of arriving at an Emergency Department (ED). Concerns over the number of cancelled elective operations at trusts, as a result of increasing emergency pressures, have also been highlighted [5]. There is increasing pressure for NHS services to return to a 95% adherence of the four-hour target as part of the NHS England 'Next steps on the NHS five year forward view'[6], which has received some criticism [7]. Deliverables have been set which include looking to increase 'front door streaming', improving patient flow and reducing delayed transfers of care. There is currently uncertainty around the impact these interventions will have and if they are likely to result in a return to the performance targets expected.

There are several analyses which aim to understand the causes for ED performance decline and increasing pressure on acute services in England over recent years. Some analyses and commentary have suggested that high rates of bed occupancy and delayed transfers of care within trusts could be increasing the pressure on acute services, and in some cases may lead to increased waiting times in ED departments [3,8–11]. However, there is currently limited peer-reviewed statistical evidence showing the relationship between these factors and routinely collected measures of pressure on acute trusts. One of the few peer-reviewed works that has been published [12] examined data over a single two-week period in 2002 and used linear regression analysis to show a relationship between hospital bed occupancy and four-hour target performance. The study is univariate however and is limited to only a small time-window, hence it only provides a limited amount of understanding of the contributing factors to four-hour target performance. Other quantitative studies investigating the cases and consequences around waiting times in EDs [13–22] have looked at data which is not openly published and often pertains only to a small number or single hospital not necessarily located in the UK. Many of these studies also only look at the ED in isolation. These studies have limited scope in explaining the relative importance of different factors affecting growing emergency pressures on hospitals in England.

A systematic quantitative study is required which looks at the patient flow factors across trusts to understand the factors that trusts can modify which could improve performance and care. Greater understanding of the factors may allow appropriate targeting of resources tailored to trusts, rather than a suite of measures which are expected to be implemented across all providers. The aim of this work is to investigate the relative impact of the commonly highlighted variables: bed occupancy, delayed transfers of care, and other routinely measured operational factors, on the four-hour target as well as on cancelled elective operations across Acute trusts in England. This will provide evidence of the relative importance of each of these factors as well as how these dependencies have changed over time. It will also demonstrate how routinely collected and openly reported data can be utilised in a statistically robust way to understand more about how pressures on NHS services are changing.

Simplified high level system flow

Figure 1 shows a simplified hospital trust system and the patient flows through it. Patients attending ED will either be admitted as an inpatient to the trust or will be discharged from the ED. Patients may only be admitted from ED to the inpatient provision if there is space available (usually in the form of a bed). Space will only become available as inpatients are discharged or transferred from the trust to: home, social care provision or another trust. Additionally, there is pressure to admit from patients undergoing elective (non-emergency) procedures. Any of these patient flows could be a bottleneck which can result in a deterioration of trust performance measures.

Two measures of the pressure a trust is facing are breached attendances and cancelled elective operations; these are commonly thought to be a measure of pressure on EDs and inpatient provision respectively. There are several factors, related to patient flow, which may provide insight into the pressures that trusts are currently facing. Those which will be investigated in this study are related to: bed occupancy, which is reported both in day and night beds; day to night bed ratio, indicating the split between bed types in a trust; delayed transfers of care; conversion ratio, the proportion of patients attending ED who are admitted; casemix ratio, indicating the split between emergency

For peer review only - http://bmjopen.bmj.com/site/about/guidelines.xhtml

and elective admissions; and the number of admissions per bed within a trust. Figure 1 highlights these measures around the area they are likely to affect the hospital system most acutely.

Figure 1: a simplified trust system broadly illustrating patient flow.

For peer review only

Internal workings of an ED

The efficiency of internal ED micro-flow has been analysed extensively using modelling and simulation[23–25]. This, for example, has explored the use of fast tracks for minor injuries or patient 'streaming' (now common place in the England[26]; prioritisation by acuity[17]; and workforce scheduling and resourcing[27]. Processes of patient flow through ED and into trusts have also been shown to vary considerably between sites [17]. However, our unit of analysis is the hospital and its effects on four-hour performance.

METHODS

Data collation

A data set was collated from published open-data hosted on the NHS England Statistics website [28].These data sets included data for all NHS trusts in England, as well as minor injury units and walk-in-centres. At the time of collation data was available from quarter 2, 2011 until quarter 4, 2016 (calendar year) for the statistical reports entitled:

- Emergency department attendances and emergency admissions
- Bed availability and occupancy
- Cancelled elective operations
- Delayed transfers of care
- Hospital activity

The data source is created from each NHS organisation routinely reporting their own counts of activity, which NHS England collate and publish. This is a separate source from the Secondary Uses Service or Hospital Episodes Statistics. Full definitions of the indicators and the rules for submission by the providers are available from NHS England [28].

Trust filtering

For peer review only - http://bmjopen.bmj.com/site/about/guidelines.xhtml

The collated dataset was filtered to only include NHS trusts in England defined as: Small, Medium, Large Acute and Teaching. These trusts are the organisations who have seen the greatest reduction in the four-hour target. Mental health trusts, acute specialist trusts, walk in centres, practices, health centres, out-of-hours services and treatment centres were all excluded from the analysis. The definitions for NHS trust types are available online [29].

Study variables

The collated data contained counts of events for each NHS trust in England, which were converted into a proportion or a ratio using an appropriate denominator specific to the same trust for each time-period. For example: the 'number of attendances in ED lasting greater than four-hours' was divided by 'total number of ED attendances' of the period at that trust, and the 'number of beds occupied' were divided by 'total number of beds'. This created variables, which allowed useful comparisons between trusts of different sizes and activity levels. A summary of the variables investigated in this study are included in table 1, along with information on how they were calculated and a description of how they should be interpreted. The variables in this study were created from aggregating quarterly data for each year between (2012-2016) and relate to those in figure 1.

For peer review only

Table 1: Variables included in the study.

Variables	Type	Numerator	Denominator	Units	Transformation applied?	Interpretation
Breached attendances	Outcome	Number of ED attendances greater than 4 hours	Number of ED attendances	-	-	Proportion of ED attendances waiting >4hrs.
Cancelled electives	Outcome	Number of cancelled elective operations	Number of elective admissions	Operations per admission	log	Ratio of cancelled elective operations to elective admissions. In absence of number of planned elective operations this is the most suitable denominator.
Empty day beds	Explanatory	Number of unoccupied day beds	Number of day beds	-	Categorised (5)	Ratio of unoccupied day beds to total number of day beds.
Empty night beds	Explanatory	Number of unoccupied night beds	Number of night beds	-	log	Ratio of unoccupied night beds to total number of night beds.
Delayed transfers	Explanatory	Number of bed days taken by delayed transfers	Number of night beds	10 bed-days	-	The number of bed-days lost to delayed transfers for each night bed at a trust, over the course of a year.
Day to night bed ratio	Explanatory	Number of day beds	Number of night beds	-	-	Ratio of total day beds to total night beds.
ED conversion ratio	Explanatory	Number of emergency admissions via ED	Number of attendances at ED	-	-	Ratio of ED admissions to attendances. Often commonly referred to as 'Conversion ratio'.
Admission casemix ratio	Explanatory	Number of non-elective (emergency) admissions	Number of elective admissions	-	log	Proportion of admissions that are emergency or ratio of emergency to elective admissions.
Emergency admission/ bed ratio	Explanatory	Number of non-elective (emergency) admissions	Number of day and night beds	10 Admissions	-	Number of emergency admissions per bed over the course of a year.

For peer review only - http://bmjopen.bmj.com/site/about/guidelines.xhtml

Variable distributions and transformation

Some variables were transformed to provide a normal distribution for regression model fitting. After conversion into proportions/ratios, non-normal variables were transformed using a natural log function (cancelled electives, empty night beds, admission casemix ratio). One variable (empty day beds) contained zero values and hence a log transformation was not appropriate. This variable was categorised. Bin edges were determined to provide approximately equal numbers of samples in each group. Where this was conducted bin sizes were created based on data across all years of study in order to provide consistent transformation.

Bias

Some missing data was found where trusts had not submitted data. For each year studied no more than 4% of trusts were found to have missing data for at least one variable; therefore the maximum percentage of missing data points for any regression was less than 4%. Our initial protocol intended to blind the variables throughout the analysis, however variables were un-blinded part way through the study as it was decided that greater contextual understanding of the problem was required to fully develop the analysis.

Within Trusts the reported activity is split into types 1,2 &3. Type 2 and type 3 EDs are defined by NHS England as: Minor Injury Units, Eye Casualties, Urgent Care Centres and Walk-In Centres. At some trusts these services are co-located at the same hospital site and so these could not be excluded from the analysis as the attendances contribute to hospital patient flow. It is possible that some four-hour target variation across Trusts may be due to larger volumes of patients attending type 2 & 3 units for minor injuries. As Trusts are often measured by their four-hour target performance based on all ED types, and the focus of this work is on patient flow across Trusts, the analysis conducted in this paper includes attendances at all types of department within each Trust.

Statistical methods

Univariate ordinary least squares linear regression was conducted using breached attendances as the outcome variable against each of the explanatory variables. To ascertain how the importance of each variable has changed over time the regression analysis was performed for each year separately, in a series of cross-sectional studies. As this method could present a statistical problem of multiple comparisons, undue emphasis was not placed on statistical significance tests. Results are presented as an exploratory study, showing the regression coefficients, associated confidence intervals and coefficient of determination values in each case. Only consistent associations which are of clinical importance are highlighted in the discussion. Multivariate regression was also performed to ascertain the relative importance of each predictor variable on breached attendances when combined into a single model. A model containing all predictor variables which have been highlighted of clinical importance, and those which showed considerable association strength to the outcome variable in the univariate regression analysis, was created to provide some understanding of the interaction between predictor variables. The univariate and multivariate models' residuals were checked visually for normality and homogeneity. Influential outliers with high leverage were also investigated using Cook's distance, but not removed. Abnormalities are reported in the results section. The same method of analysis was repeated using cancelled elective operations as the explanatory variable. Examples of the plots used to check the models are provided in Appendix A.

All the analysis conducted in this work was completed with the python language (version 3.6.0, www.python.org) using the: Statsmodels (version 0.8.0, www.statsmodels.org) and Pandas (version 0.19.2, pandas.pydata.org) libraries. This was done in an Anaconda environment (www.anaconda.com) utilising Jupyter notebooks (www.jupyter.org).

RESULTS

In 2012 there were 254 organisations in England reporting at least one of the variables investigated in this study (226 in 2016). Figure 2 shows the distribution of four-hour target breaches by organisation type. The acute and teaching trusts are observed to have higher proportions of breaches, with increases between 2012 and 2016. Most non-acute

For peer review only - http://bmjopen.bmj.com/site/about/guidelines.xhtml

and specialist trusts still conform to the four-hour target in 2016. Hence the former group were the focus of this study. The number of trusts reduced to 142 when applying the criteria of the organisation type for the study (135 in 2016). Missing data reduced the number of trusts for some variables to 136 (131 in 2016).

Figure 2: the distribution of four-hour target breaches by organisation type for 2012 and 2016.

How variables have changed over time

Table 2 quantifies changes of each variable between 2012-2016. Between 2012 and 2016 the median values of breached attendances and cancelled elective operations has increased. The spread of the values has also increased in the time period in both cases, however more noticeably by over three times in the case of 'breached attendances'. The most noticeable increases occurred between 2015 and 2016 in both cases. There has been a decline the proportion of empty night beds between 2015 and 2016 (it should be noted that this variable is the reverse of occupancy; 'empty beds' = 1 – 'occupancy'). This reflects the historical reduction in night beds which has been noted elsewhere [10]. Between 2012 and 2016 there has been a noticeable increase in delayed transfers and emergency admission to bed ratios across trusts. Median bed-days and IQR lost due to delayed transfers has doubled over the period. Less prominent increases are observed in: empty day beds, day to night bed ratio and admission casemix ratio over this period. Although there are some fluctuations in ED conversion ratio, there is no overall change across the period.

Table 2: Changes in distribution of explanatory variables between 2012 and 2016.

Variable		2012	2013	2014	2015	2016
Breached attendances	median	0.05	0.05	0.06	0.07	0.13
	IQR	0.02	0.02	0.03	0.06	0.07
	5-95%	0.05	0.07	0.10	0.11	0.16
Cancelled electives	median	0.012	0.013	0.013	0.013	0.015
	IQR	0.007	0.008	0.010	0.010	0.011
	5-95%	0.019	0.019	0.020	0.021	0.026
Empty day beds	median	0.10	0.10	0.09	0.11	0.11
	IQR	0.17	0.17	0.17	0.17	0.21
	5-95%	0.38	0.37	0.43	0.40	0.37
Empty night beds	median	0.13	0.13	0.13	0.13	0.11
	IQR	0.08	0.08	0.09	0.08	0.07
	5-95%	0.16	0.17	0.18	0.18	0.14
Delayed transfers	median	0.73	0.77	0.91	1.04	1.36
	IQR	0.76	0.71	0.75	1.05	1.18
	5-95%	1.56	1.72	1.88	2.25	2.99

For peer review only

For peer review only - http://bmjopen.bmj.com/site/about/guidelines.xhtml

Day to night bed ratio	median	0.10	0.10	0.10	0.11	0.11
	IQR	0.07	0.07	0.07	0.08	0.08
	5-95%	0.14	0.17	0.17	0.18	0.17
ED conversion ratio	median	0.22	0.23	0.23	0.24	0.23
	IQR	0.09	0.09	0.10	0.09	0.08
	5-95%	0.19	0.19	0.19	0.19	0.19
Admission casemix ratio	median	1.15	1.13	1.16	1.17	1.19
	IQR	0.39	0.51	0.45	0.41	0.40
	5-95%	1.12	1.21	1.59	1.55	1.67
Emergency admission/ bed ratio	median	4.42	4.47	4.62	4.76	4.93
	IQR	0.94	1.10	1.05	0.99	1.08
	5-95%	2.43	2.45	2.61	2.65	2.69

Breached Attendances: Univariate regression analysis

Table 3 gives the results of the univariate regression models using breached attendances as the outcome variable. Night bed emptiness and ED conversion ratio showed respectively positive and negative associations, with breached attendances, consistently for each year of study. For night bed emptiness in 2012 the R^2 value 0.10 is of similar magnitude to a previous study [12], however in subsequent years is observed to reduced. The day to night bed ratio showed a negative relationship with trust breaches, which increased in strength during 2015-2016; it also shows an increasing ability to account for breaches (increasing R value between 2014-2016). The delayed transfers variable showed little evidence of a linear association with breached attendances between 2012-2015.

Table 3: univariate regression for the breached attendances outcome variable. Note: all results were based on data from 131 or more trusts (* indicates non-normality, heteroskedasticity or influential outlier in regression; 'Reg coef' is the regression coefficient, β).

Variable	Parameter	Year				
		2012	2013	2014	2015	2016
Empty day beds	R^2	0.02	0.01	0.01*	0.00*	0.00
	Reg coef (95% CIs)	-0.001 (-0.003,0.001)	-0.001 (-0.004,0.002)	0.001 (-0.002,0.005)	0.001 (-0.003,0.006)	0.002 (-0.004,0.008)
	p	0.15	0.43	0.4	0.54	0.6
Empty night beds	R^2	0.10*	0.09	0.07	0.05	0.06*
	Reg coef (95% CIs)	-0.012 (-0.019,-0.006)	-0.015 (-0.022,-0.007)	-0.017 (-0.027,-0.006)	-0.017 (-0.031,-0.004)	-0.025 (-0.042,-0.008)
	p	<0.01	<0.01	<0.01	0.01	<0.01
Delayed transfers	R^2	0.04*	0.02	0.01*	0.01	0.04
	Reg coef (95% CIs)	0.006 (0.001,0.011)	0.005 (-0.001,0.011)	0.003 (-0.004,0.011)	0.003 (-0.005,0.011)	0.011 (0.002,0.020)
	p	0.01	0.13	0.36	0.43	0.02
Day to night bed ratio	R^2	0.03	0.02	0.01	0.04	0.05
	Reg coef (95% CIs)	-0.058 (-0.118,0.002)	-0.066 (-0.138,0.006)	-0.058 (-0.156,0.040)	-0.146 (-0.264,-0.027)	-0.218 (-0.378,-0.058)
	p	0.06	0.07	0.25	0.02	0.01
ED conversion	R^2	0.07	0.05	0.05*	0.05	0.03
	Reg coef (95% CIs)	0.071 (0.027,0.115)	0.082 (0.025,0.140)	0.114 (0.033,0.195)	0.143 (0.039,0.248)	0.144 (0.000,0.288)
	p	<0.01	0.01	0.01	0.01	0.05
Admission	R^2	0.00	0.00	0.00	0.00*	0.00

For peer review only - http://bmjopen.bmj.com/site/about/guidelines.xhtml

casemix	Reg coef (95% CIs)	0.002 (-0.007,0.010)	0.001 (-0.010,0.012)	-0.002 (-0.016,0.012)	0.007 (-0.012,0.025)	-0.001 (-0.027,0.024)
	p	0.70	0.86	0.77	0.47	0.93
Emergency admission to bed ratio	R^2	0.01	0.02	0.01*	0.04	0.01
	Reg coef (95% CIs)	0.002 (-0.001,0.006)	0.003 (-0.001,0.008)	0.003 (-0.002,0.009)	0.009 (0.001,0.017)	0.005 (-0.005,0.015)
	p	0.21	0.14	0.25	0.03	0.33

Breached attendances: Multivariate regression analysis

Table 4 shows the results of the multivariate regression for 2012 and 2016. In 2012 empty night beds and ED conversion ratio variables were both statistically significant and the model was able to explain 19% of the variation in the breached attendances variable. In 2016 the results of the multivariate model show only day to night bed ratio to be statistically significant in predicting breached attendances. The R^2 value indicates that only 12% of the variation in breached attendances can be accounted for with the parameters investigated in this study in 2016. When applying the multivariate model for other years a reduction in the importance of empty night beds and ED conversion ratio, and an increase in importance of day to night bed ratio was steadily observed between 2012-2016.

Table 4: multivariate regression model output for breached attendances as explanatory variable (R^2 = 0.19 in 2012, R^2 = 0.1 in 2016).

	2012			2016		
Variable	Regression coefficient	p	95% CIs	Regression coefficient	p	95% CIs
Empty night beds	-0.010	<0.01	-0.016 -0.004	-0.016	0.09	-0.034 0.003
Delayed transfers	0.004	0.12	-0.001 0.008	0.006	0.24	-0.004 0.015
Day to night bed ratio	-0.045	0.12	-0.102 0.012	-0.169	0.04	-0.330 -0.008
ED conversion ratio	0.068	<0.01	0.025 0.112	0.104	0.17	-0.044 0.252

Cancelled elective operations: Univariate regression analysis

Table 5 gives the results of the univariate regression models using cancelled elective operations as the outcome variable. ED conversion ratio, admission casemix ratio and delayed transfers showed a positive relationship with cancelled elective operations. Between 2014-2016 the variables ED conversion ratio and admission casemix ratio show increasingly positive associations with cancelled elective operations. These variables also demonstrate a relatively high ability to explain the outcome variable in comparison to the other variables in the study (R^2 values 0.07 & 0.11 respectively in 2016). The delayed transfers variable is observed to increase in importance between 2013-2016. The other variables included in the study did not show evidence of any clear association with cancelled elective operations.

Table 5: univariate regression output for the cancelled elective operations outcome variable (* indicates non-normality, heteroskedasticity or influential outlier in regression; 'Reg coef' is regression coefficient, β).

Variable	Parameters	Year				
		2012	2013	2014	2015	2016
Empty day beds	R^2	0.00	0.00	0.00	0.00	0.00
	Reg coef	0.009	0.009	0.008	-0.014	-0.003

For peer review only - http://bmjopen.bmj.com/site/about/guidelines.xhtml

	(95% CIs)	(-0.047,0.066)	(-0.046,0.064)	(-0.051,0.066)	(-0.079,0.051)	(-0.069,0.062)
	p	0.75	0.75	0.8	0.68	0.92
Empty night beds	R^2	0.01	0.02	0.00	0.00	0.02
	Reg coef	-0.135	-0.130	-0.087	-0.066	-0.149
	(95% CIs)	(-0.328,0.059)	(-0.307,0.047)	(-0.273,0.099)	(-0.260,0.129)	(-0.336,0.039)
	p	0.17	0.15	0.36	0.51	0.12
Delayed transfers	R^2	0.01	0.00*	0.01*	0.03*	0.03
	Reg coef	0.087	0.040	0.065	0.108	0.103
	(95% CIs)	(-0.064,0.237)	(-0.097,0.178)	(-0.063,0.192)	(-0.007,0.222)	(0.004,0.201)
	p	0.26	0.56	0.32	0.07	0.04
Day to night bed ratio	R^2	0.01	0.00	0.00	0.00	0.00
	Reg coef	1.067	0.589	0.245	0.255	-0.677
	(95% CIs)	(-0.733,2.868)	(-0.997,2.175)	(-1.410,1.900)	(-1.444,1.954)	(-2.437,1.082)
	p	0.24	0.46	0.77	0.77	0.45
ED conversion	R^2	0.05	0.02	0.07	0.06	0.07
	Reg coef	1.728	1.020	2.275	2.108	2.419
	(95% CIs)	(0.402,3.055)	(-0.288,2.327)	(0.914,3.637)	(0.627,3.589)	(0.901,3.937)
	p	0.01	0.13	<0.01	<0.01	<0.01
Admission casemix	R^2	0.02	0.05	0.13	0.12*	0.11
	Reg coef	0.240	0.318	0.507	0.524	0.518
	(95% CIs)	(-0.016,0.496)	(0.082,0.553)	(0.288,0.726)	(0.275,0.772)	(0.258,0.778)
	p	0.07	<0.01	<0.001	<0.001	<0.001
Emergency admission to bed ratio	R^2	0.00	0.00	0.00	0.00	0.00
	Reg coef	0.029	0.010	0.009	-0.025	-0.033
	(95% CIs)	(-0.077,0.136)	(-0.087,0.108)	(-0.089,0.107)	(-0.136,0.087)	(-0.143,0.077)
	p	0.58	0.84	0.85	0.66	0.56

Cancelled electives: Multivariate regression analysis

Table 6 shows a summary of the variables included in the multivariate linear regression model using cancelled elective operations as an explanatory variable in 2012 and 2016. In 2012 the only statistically significant variable related to cancelled elective operations was ED conversion ratio, which had a positive association. In 2016 the results indicate that the statistically significant variables in explaining variation were ED conversion ratio and admission casemix ratio. Both of these variables were observed to be statistically significant in the model for year between 2014-2016. Overall the model was able to account for 17% of the variation in cancelled elective operations in 2016, which demonstrates an increasing ability to determine cancelled elective operations over the study period (increasing from 7% in 2012).

Table 6: multivariate regression model output for cancelled electives as explanatory variable (R^2 = 0.07 in 2012; R^2 = 0.17 in 2016).

	2012			2016		
Variable	Regression coefficient	p	95% CIs	Regression coefficient	p	95% CIs
Delayed transfers	0.067	0.37	-0.081 0.215	0.070	0.15	-0.025 0.164
ED conversion ratio	1.625	0.02	0.302 2.949	1.881	0.01	0.385 3.377
Admission casemix ratio	0.225	0.08	-0.027 0.477	0.483	<0.001	0.230 0.736

For peer review only

DISCUSSION

Summary of findings

The intra-hospital patient flow variables with greatest association with better acute trust four-hour target performance in England were found to have varied over the period 2012-2016. The main variables of interest in explaining performance in 2012 were found to be the proportion of empty night beds and ED conversion ratio. These variables were seen to have reducing importance over the subsequent years in the multivariate model. The day to night bed ratio variable was observed to have increased in importance between 2012 and 2016, and in the years 2015-2016 it was the most important patient flow factor in explaining four-hour target performance in the multivariate model. The results also show that intra-hospital patient flow is only responsible for explaining some of the variation in four-hour target performance, and between 2012-2016 demonstrated a reducing ability to explain this variation (R^2 value of model reducing from 0.19 to 0.12). There was limited evidence of a clear association between delayed transfers of care and four-hour target performance, either in the univariate or multivariate model results.

The main intra-hospital patient flow variables associated with higher levels of cancelled elective operations at acute trusts between 2012-2016 were ED conversion ratio and the admission casemix ratio. Between 2012-2016 the importance of the admission casemix ratio is observed to have increased beyond that of ED conversion ratio. The ability of the multivariate model to explain variation in cancelled elective operations has increased between 2012 and 2016 (R^2 value of model increasing from 0.07 to 0.17), indicating that the emergency workload that trusts face are becoming an increasingly important factor in elective procedures being cancelled in England.

How important is intra-hospital flow to the four-hour target?

The ability of the multivariate model to explain only 12% of the variation in four-hour target in 2016 (which has reduced since 2012 from 17%) indicates that there are other factors that are increasingly important in determining four-hour performance. By comparison, another multivariate model could predict only 6.8% variation in four-hour target (using patient demographics and satisfaction with GP service rates) [22]. There are a number of factors which may affect ED processes and performance measures: macro or intra-hospital flow (i.e. patient flows across the whole trust, such as those investigated in this study); micro-flow factors within departments/wards (i.e. staffing[4,17], work flows[26,30], access to diagnostics[17]); population factors (i.e. age, sex, deprivation[22,31], access to GP/community/social care services[22,32]); noise (i.e. recording errors, reporting differences[10], 'gaming' of four-hour target[5,17]). If the mechanisms of pressures facing acute trusts are to be understood more fully, future work is required to quantify the relative effects of each of these factors on trust performance measures.

Bed occupancy

In our univariate analysis night-bed occupancy was consistently associated with 4-hr performance (2012-2016). This is a result consistent with queuing theory. That is, if trusts do not provide adequate bed 'buffer' capacity to cope with the peaks of emergency admission demand then ED performance will decline. As such trusts must continue to focus on innovations to reduce night-bed occupancy. Targets such as 85% occupancy have been proposed elsewhere [33] although this seems unrealistic given in 2012 and 2016 respectively only 39% and 25% of trusts attained this level of night-bed occupancy (or lower).

Day to night bed ratio and using day beds as 'buffers'

Our results show that that trusts with higher ratio of day to night bed capacity were more likely to have higher four-hour performance in 2016. It has been highlighted that admission and discharge patterns through trusts have peaks at different times of day [9,11]. Hence an explanation for this result is that day-bed capacity can be used flexibly as a temporary 'buffer' for patient admission whilst the discharge of patients from the trust catches up during the day. Day beds are defined as consultant-led beds that are closed overnight [28], hence patients are not able to occupy them for long periods as in the case of night beds, and the occupancy of such wards/areas will be low at the

For peer review only - http://bmjopen.bmj.com/site/about/guidelines.xhtml

For peer review only

beginning of each day to allow admissions. It is possible that more trusts are taking advantage of this flexibility as the day to night bed ratio of trusts in England has increased between 2012-2016 (see table 2). This may be in a response to trusts operating at greater levels of occupancy (also see table 2) towards the end of this period, and requiring a way of ensuring bed 'buffers' are available to allow patients to be admitted more promptly. We note that if occupancy can be reduced this may remove the need to use day beds as internal buffers.

Delayed transfers

Our expectations were that delayed transfers of care would be a strong predictor of four-hour performance. Our results do not support this commonly held assumption. This suggests that hospital initiatives to reduce delayed transfers of care may not yield the expected benefits for ED waiting times. We in no way suggest that initiatives to reduce delayed transfers are not worthwhile, as expedited transfers to appropriate care such as rehabilitation or social care have clear clinical and quality of life benefits for patients. We do note the strong association between in-patient bed occupancy (night-bed occupancy) and delayed transfers of care (Spearman's rank correlation in 2016 = -0.27, p = 0.002). However, it is clear that a focus on only delayed transfers will not reduce occupancy to a level that is sufficient to release the pressure on EDs; more holistic approaches to reducing bed occupancy may be required.

'Clinical streaming' and conversion ratio

'Clinical streaming' in ED aims to triage patients within 15 minutes of their arrival, and refer patients to other appropriate services [34]. This aims to: reduce the load on EDs by only treating patients who cannot be treated by other services, and provide better patient flow within EDs by prioritising different routes of care suited to patient needs. With regard to our study focussing on patient flow it is plausible that clinical streaming may affect ED conversion ratio as there is currently some limited evidence that earlier review by senior clinicians may reduce avoidable admissions [35]. Our results show that ED conversion ratio was important in explaining some variation in four-hour performance and cancelled elective operations, however conversion ratios of trusts in England have not changed noticeably between 2012 -2016 (see table 2) whilst some trusts are already known to have introduced clinical streaming [17]. It is currently unclear if there would be significant changes to trust conversion ratios with the proposed roll-out of clinical streaming [6] by NHS England. More research is needed to understand if clinical streaming impacts on patient flow in a positive manner.

Strengths and weaknesses of study

This study is the first to examine in detail the in-hospital factors that influence ED four-hour performance. We have analysed the change in the importance of common explanatory theories of hospital flow bottlenecks across five years using recent openly published data. We believe the study provides a simple and transparent analysis which can contribute to the discussion around the causes of decline in ED four-hour performance targets.

Trust four-hour target reporting is known to be affected by 'gaming' [5], which may introduce extra variability into the relationships under investigation. However, this is the measure which trusts are judged and funded, therefore it is argued that it is suitable for use within this study. The quality of data recording by trusts of the 'delayed transfers' variable has been reported to be questionable [10] and under-reported [36], however there is currently little published evidence around this issue. The data quality may explain the uncertainly in the importance of this variable over time in our analysis.

The R^2 values found, although higher than other multivariate models predicting the same outcome [22], indicate that there is limited ability of the models to predict the outcome variables. It is possible that more complex statistical methodologies could provide a greater predictive capability. For example, it is plausible that extreme values in some of the variables investigated may lead to changes in another, and otherwise may have little impact. This may, for example, explain our results for Delayed Transfers of Care. Hence one possible example could be the use of generalised additive models, which would be able to account for possible non-linear relationships between the rates and ratios investigated in this study. It is also possible there may be trusts where specific variables impact on outcome measures but are not relevant to other trusts. It may be possible to use mixed models to investigate this further. A detailed longitudinal analysis could also be of benefit to provide greater understanding of the dependence

For peer review only - http://bmjopen.bmj.com/site/about/guidelines.xhtml

of the variables over the study period. The data and open-source analysis code are supplied with this publication to allow the further development of this work and the open development of more complex models.

This study focusses on the macro-flow factors across trusts. Future work including the relative importance of the macro flow, micro flow, population factors and noise would be of value to assess the different pressures trusts face and aid in the targeting of service re-configurations.

FOOTNOTES

We thank the NHS England Statistics Unify2 team, for help in accessing and understanding the open data utilised in this study.

Transparency: The lead author (the manuscript's guarantor) affirms that the manuscript is an honest, accurate, and transparent account of the study being reported; that no important aspects of the study have been omitted; and that any discrepancies from the study as planned (and, if relevant, registered) have been explained. The authors attest that we have obtained appropriate permissions and paid any required fees for use of copyright protected materials.

Datasharing: All code required to re-produce this work is available as supplementary files to the publication. The raw collated quarterly count data as well as the python code to produce the year-aggregated variable data used in this study has also been made available [37] in the hope that other researchers will be easily able to reproduce this analysis, and allow further analysis to be conducted to test other research hypotheses.

Ethics: This study was reviewed by University of Southampton ERGO ethics committee (reference 25957).

Contributors: RG created the initial research question. BK contributed to the initial design of the work and was responsible for the protocol/ethics, data acquisition and collation, coding, analysis and interpretation of results. DC was responsible for the statistical methodology and interpretation of results. TM was involved throughout the study with guidance in the design, methodology and interpretation of results. All authors were involved in the development and review of the manuscript.

Competing interests: All authors have completed the ICMJE uniform disclosure form at www.icmje.org/coi_disclosure.pdf and declare: no support from any organisation for the submitted work; no financial relationships with any organisations that might have an interest in the submitted work in the previous three years; no other relationships or activities that could appear to have influenced the submitted work.

Funding: This article presents independent research funded by the National Institute for Health Research (NIHR) Collaboration for Leadership in Applied Health Research and Care (CLAHRC) Wessex. The views expressed in this publication are those of the author(s) and not necessarily those of the National Health Service, the NIHR, or the Department of Health.

Copyright: The Corresponding Author has the right to grant on behalf of all authors and does grant on behalf of all authors, an exclusive licence (or non exclusive for government employees) on a worldwide basis to the BMJ Publishing Group Ltd to permit this article (if accepted) to be published in BMJ editions and any other BMJPGL products and sublicences such use and exploit all subsidiary rights, as set out in our licence.

REFERENCES

1. Blunt I, Edwards N, Merry L. What's behind the A&E "Crisis"? The Nuffield Trust. 2015.

2. Appleby J. Nuffield Winter Insight Briefing 1: Winter beds pressures. The Nuffield Trust. 2016.

3. Fisher E, Dorning H. Winter pressures: what's going on behind the scenes ? The Nuffield Trust. 2016.

For peer review only - http://bmjopen.bmj.com/site/about/guidelines.xhtml

For peer review only

4. House of Commons Health Committee. Urgent and emergency services. 2013.

5. House of Commons Health Comittee. Winter pressure in accident and emergency departments. 2016.

6. NHS England. Next Steps on the NHS Five Year Forward View. 2017.

7. Oliver D. Reducing acute admissions? a timely reality check. BMJ. 2017;357:j2082.

8. Monitor. A&E delays : Why did patients wait longer last winter? 2015.

9. Karakusevic S. Understanding patient flow in hospitals. The Nuffield Trust. 2016.

10. British Medical Association. State of the health system Beds in the NHS: England. 2017.

11. Allder S, Silvester K, Walley P. Understanding the current state of patient flow in a hospital. Clin Med (Lond). 2010;10(5):441–4.

12. Cooke MW, Wilson S, Halsall J, Roalfe A. Total time in English accident and emergency departments is related to bed occupancy. Emerg Med J. 2004;21(5):575–6.

13. Mustafa F, Gilligan P, Obu D, O'Kelly P, O'Hea E, Lloyd C, et al. "Delayed discharges and boarders": a 2-year study of the relationship between patients experiencing delayed discharges from an acute hospital and boarding of admitted patients in a crowded ED. Emerg Med J. 2016;33:636–40.

14. Sun BC, Laurie A, Prewitt L, Fu R, Chang AM, Augustine J, et al. Risk-Adjusted Variation of Publicly Reported Emergency Department Timeliness Measures. Ann Emerg Med. 2016;67(4):509–516.e7.

15. Rathlev NK, Chessare J, Olshaker J, Obendorfer D, Mehta SD, Rothenhaus T, et al. Time Series Analysis of Variables Associated With Daily Mean Emergency Department Length of Stay. Ann Emerg Med. 2007;49(3):265–71.

16. Silvester K, Harriman P, Walley P, Burley G. Does process flow make a difference to mortality and cost? An observational study. Int J Health Care Qual Assur. 2014;27(7):616–32.

17. Pinkney J, Rance S, Benger J, Brant H, Joel-Edgar S, Swancutt D, et al. How can frontline expertise and new models of care best contribute to safely reducing avoidable acute admissions? A mixed-methods study of four acute hospitals. Health Services and Delivery Research. 2016;4(3).

18. Blom MC, Jonsson F, Landin-Olsson M, Ivarsson K. Associations between in-hospital bed occupancy and unplanned 72-h revisits to the emergency department: a register study. Int J Emerg Med. 2014;7:25.

19. Graham J, Aitken ME, Shirm S. Correlation of measures of patient acuity with measures of crowding in a pediatric emergency department. Pediatr Emerg Care. 2011;27(8):706–9.

20. Harris A, Sharma A. Access block and overcrowding in emergency departments: an empirical analysis. Emerg Med J. 2010;27(7):508–11.

21. Vermeulen MJ, Ray JG, Bell C, Cayen B, Stukel TA, Schull MJ. Disequilibrium Between Admitted and Discharged Hospitalized Patients Affects Emergency Department Length of Stay. Ann Emerg Med. 2009;54(6):794–804.

22. Blunt I. Focus on: A&E attendances. Quality Watch: The Health Foundation and the Nuffield Trust. 2014.

23. Mohiuddin S, Busby J, Savovi J, Richards A, Northstone K, Hollingworth W, et al. Patient flow within UK emergency departments: a systematic review of the use of computer simulation modelling methods. BMJ Open. 2017;7:e015007.

24. Saghafian S, Austin G, Traub SJ. Operations research/management contributions to emergency department patient flow optimization: Review and research prospects. IIE Trans Healthc Syst Eng. 2015;5:101–23.

25. Gul M, Guneri AF. A comprehensive review of emergency department simulation applications for normal and disaster conditions. Comput Ind Eng. 2015;83:327–44.

26. Saghafian S, Hopp WJ, Oyen MP Van, Desmond JS, Kronick SL. Patient Streaming as a Mechanism for

For peer review only - http://bmjopen.bmj.com/site/about/guidelines.xhtml

Improving Responsiveness in Emergency Departments. Oper Res. 2012;60(5):1080–97.

27. Duguay C, Chetouane F. Modeling and Improving Emergency Department Systems using Discrete Event Simulation. Simulation. 2007;83(4):311–20.

28. NHS England. NHS England Statistics [Internet]. 2017 [cited 2017 May 1]. Available from: https://www.england.nhs.uk/statistics/

29. NHS Improvement. NHS trust list [Internet]. 2017 [cited 2017 May 1]. Available from: http://www.nrls.npsa.nhs.uk/EasySiteWeb/GatewayLink.aspx?alId=135142

30. Han JH, France DJ, Levin SR, Jones ID, Storrow AB, Aronsky D. The effect of physician triage on emergency department length of stay. J Emerg Med. 2010;39(2):227–33.

31. O'Cathain A, Knowles E, Maheswaran R, Pearson T, Turner J, Hirst E, et al. A system-wide approach to explaining variation in potentially avoidable emergency admissions: national ecological study. BMJ Qual Saf. 2014;23:47–55.

32. Flores-Mateo G, Violan-Fors C, Carrillo-Santisteve P, Peiro S, Argimon J-M. Effectiveness of organizational interventions to reduce emergency department utilization: A systematic review. PLoS One. 2012;7(5):1–7.

33. Bagust A, Place M, Posnett JW. Dynamics of bed use in accommodating emergency admissions: stochastic simulation model. BMJ. 1999;319:155–8.

34. NHS England. Clinical Streaming in the Accident and Emergency (A & E) Department [Internet]. 2017. Available from: https://www.england.nhs.uk/wp-content/uploads/2017/07/principles-for-clinical-streaming-ae-department.pdf

35. O'Cathain A, Knowles E, Turner J, Maheswaran R, Goodacre S, Hirst E, et al. Explaining variation in emergency admissions: a mixed-methods study of emergency and urgent care systems. Health Services and Delivery Research. 2014;2(48).

36. Nuffield Trust. What's behind delayed transfers of care? [Internet]. [cited 2017 Jan 1]. Available from: https://www.nuffieldtrust.org.uk/resource/what-s-behind-delayed-transfers-of-care#dtocs-the-tip-of-the-iceberg

37. Keogh B, Culliford D, Guerrero-Luduena R, Monks T. NHS England routinely reported measures 2011-2016 [Internet]. University of Southampton [Dataset]. 2017. Available from: doi:10.5258/SOTON/D0218

For peer review only

Figure Legends

Figure 1: a simplified trust system broadly illustrating patient flow.

Figure 2: the distribution of four-hour target breaches by organisation type for 2012 and 2016.

For peer review only - http://bmjopen.bmj.com/site/about/guidelines.xhtml

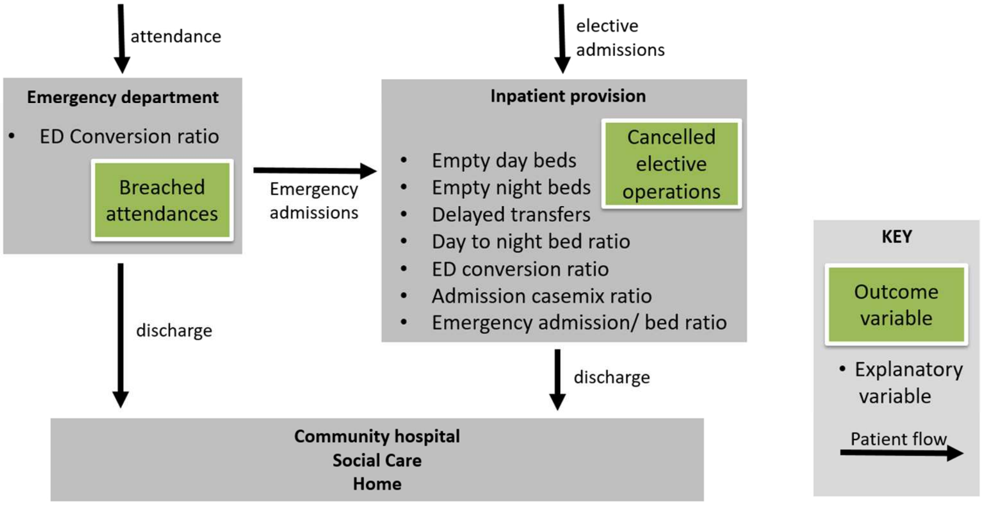

Figure 1 - a simplified trust system broadly illustrating patient flow

For peer review only

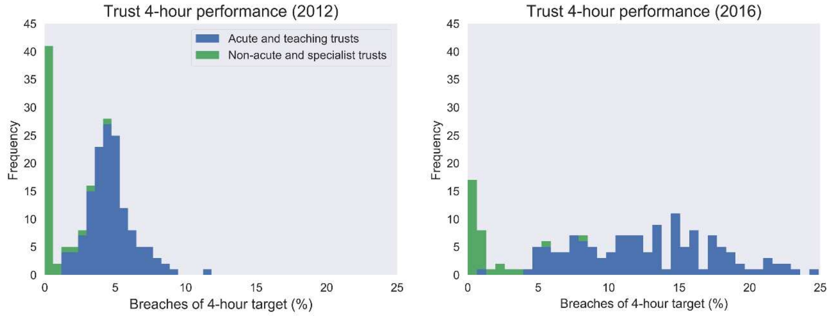

Figure 2 - the distribution of four-hour target breaches by organisation type for 2012 and 2016

For peer review only

Appendix A: Plotted outputs for sample regression case in 2016. All other models can be built, and similar plots produced, by using the data and code provided in the additional supplementary files.

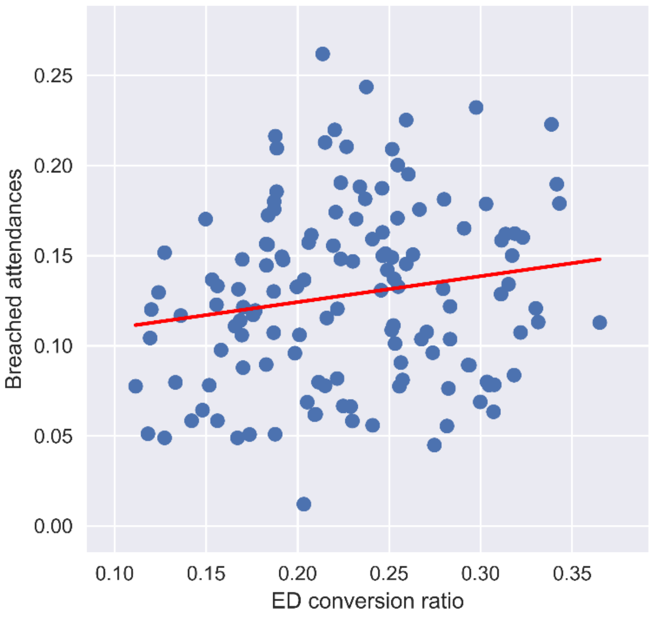

Figure A-1: Scatter plot of ‘Breached attendances’ and ‘ED conversion ratio’ with univariate linear regression model.

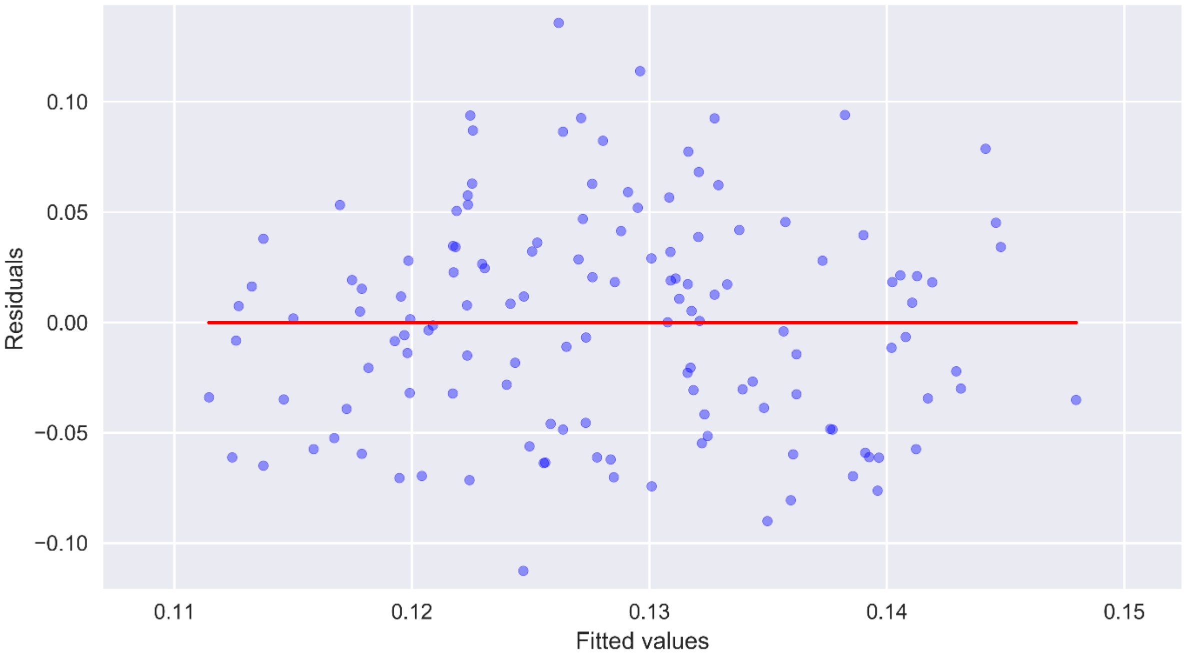

Figure A-2: Scatter plot of univariate regression residuals and fitted values from model in figure A-1.

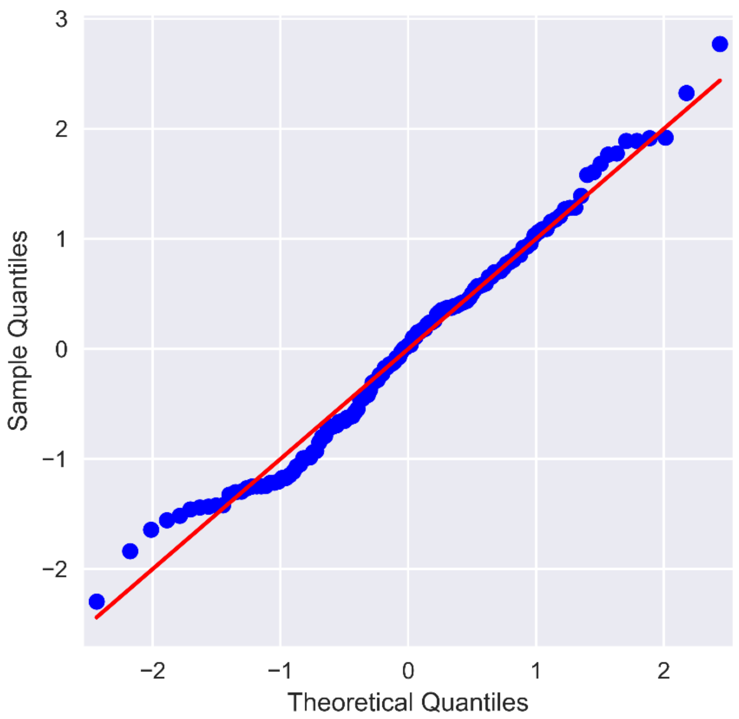

Figure A-3: QQ-plot of univariate regression model in figure A-1.

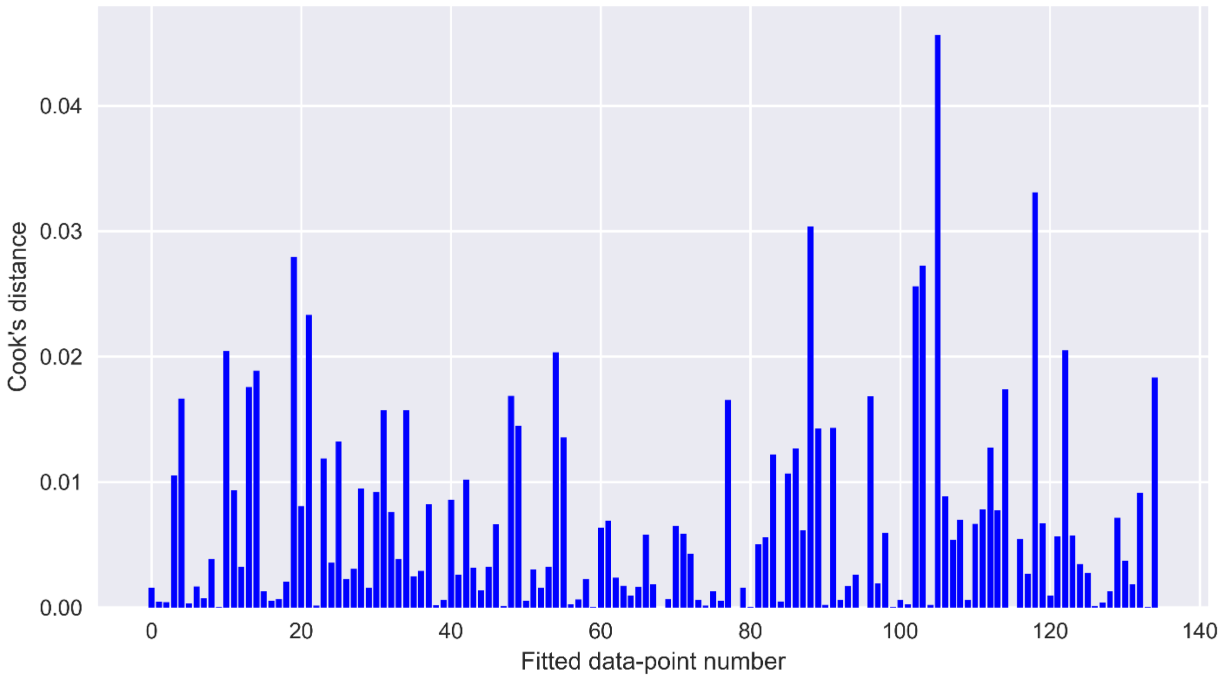

Figure A-4: Cook's distance values for each fitted data-point from model in figure A-1.

The RECORD statement – checklist of items, extended from the STROBE statement, that should be reported in observational studies using routinely collected health data.

Author note: this study is not a traditional observational study following patients (rather it follows NHS trusts), however the RECORD statement seemed most appropriate and helpful in reporting the study. As a result some of the RECORD statements were not applicable to this study. Most of the STROBE and RECORD items are located under the corresponding section titles in the manuscript but line/page numbers have been included below (all line numbers refer to authors' line numbering).

	Item No.	STROBE items	Location in manuscript where items are reported	RECORD items	Location in manuscript where items are reported
**Title and abstract**					
	1	(a) Indicate the study's design with a commonly used term in the title or the abstract (b) Provide in the abstract an informative and balanced summary of what was done and what was found	Pages 1 & 2	RECORD 1.1: The type of data used should be specified in the title or abstract. When possible, the name of the databases used should be included.<br><br>RECORD 1.2: If applicable, the geographic region and timeframe within which the study took place should be reported in the title or abstract.<br><br>RECORD 1.3: If linkage between databases was conducted for the study, this should be clearly stated in the title or abstract.	Page 2
**Introduction**					
Background rationale	2	Explain the scientific background and rationale for the investigation being reported	Page 3		
Objectives	3	State specific objectives, including any prespecified hypotheses	line 87		
**Methods**					
Study Design	4	Present key elements of study design early in the paper	See methods section starting line 121		

For peer review only

Setting	5	Describe the setting, locations, and relevant dates, including periods of recruitment, exposure, follow-up, and data collection	Line 123		
Participants	6	*(a) Cohort study* - Give the eligibility criteria, and the sources and methods of selection of participants. Describe methods of follow-up<br>*Case-control study* - Give the eligibility criteria, and the sources and methods of case ascertainment and control selection. Give the rationale for the choice of cases and controls<br>*Cross-sectional study* - Give the eligibility criteria, and the sources and methods of selection of participants<br><br>*(b) Cohort study* - For matched studies, give matching criteria and number of exposed and unexposed<br>*Case-control study* - For matched studies, give matching criteria and the number of controls per case	Line 137	RECORD 6.1: The methods of study population selection (such as codes or algorithms used to identify subjects) should be listed in detail. If this is not possible, an explanation should be provided.<br><br>RECORD 6.2: Any validation studies of the codes or algorithms used to select the population should be referenced. If validation was conducted for this study and not published elsewhere, detailed methods and results should be provided.<br><br>RECORD 6.3: If the study involved linkage of databases, consider use of a flow diagram or other graphical display to demonstrate the data linkage process, including the number of individuals with linked data at each stage.	6.1 included line 137.<br><br>6.2 & 6.3 not relevant for this study,
Variables	7	Clearly define all outcomes, exposures, predictors, potential confounders, and effect modifiers. Give diagnostic criteria, if applicable.	Table 1; page 5	RECORD 7.1: A complete list of codes and algorithms used to classify exposures, outcomes, confounders, and effect modifiers should be provided. If these cannot be reported, an explanation should be provided.	N/A for this study
Data sources/ measurement	8	For each variable of interest, give sources of data and details of methods of assessment (measurement).	Line 123		

For peer review only

		Describe comparability of assessment methods if there is more than one group			
Bias	9	Describe any efforts to address potential sources of bias	Line 7 (following table 1)		
Study size	10	Explain how the study size was arrived at	Line 30 (following table 1)		
Quantitative variables	11	Explain how quantitative variables were handled in the analyses. If applicable, describe which groupings were chosen, and why	Line 1 (following table 1)		
Statistical methods	12	(a) Describe all statistical methods, including those used to control for confounding<br>(b) Describe any methods used to examine subgroups and interactions<br>(c) Explain how missing data were addressed<br>(d) *Cohort study* - If applicable, explain how loss to follow-up was addressed<br>*Case-control study* - If applicable, explain how matching of cases and controls was addressed<br>*Cross-sectional study* - If applicable, describe analytical methods taking account of sampling strategy<br>(e) Describe any sensitivity analyses	Line 12 (following table 1)		
Data access and cleaning methods		..		RECORD 12.1: Authors should describe the extent to which the investigators had access to the database population used to create the study population.	Line 123<br><br>Line 1 (following

For peer review only

				RECORD 12.2: Authors should provide information on the data cleaning methods used in the study.	table 1)
Linkage		..		RECORD 12.3: State whether the study included person-level, institutional-level, or other data linkage across two or more databases. The methods of linkage and methods of linkage quality evaluation should be provided.	N/A for this study
**Results**					
Participants	13	(a) Report the numbers of individuals at each stage of the study (*e.g.*, numbers potentially eligible, examined for eligibility, confirmed eligible, included in the study, completing follow-up, and analysed)<br>(b) Give reasons for non-participation at each stage.<br>(c) Consider use of a flow diagram	Line 31 (following table 1)	RECORD 13.1: Describe in detail the selection of the persons included in the study (*i.e.,* study population selection) including filtering based on data quality, data availability and linkage. The selection of included persons can be described in the text and/or by means of the study flow diagram.	Line 31 (following table 1)
Descriptive data	14	(a) Give characteristics of study participants (*e.g.*, demographic, clinical, social) and information on exposures and potential confounders<br>(b) Indicate the number of participants with missing data for each variable of interest<br>(c) *Cohort study* - summarise follow-up time (*e.g.*, average and total amount)	N/A for this study, however descriptive data from line 40 (following table 1)		
Outcome data	15	*Cohort study* - Report numbers of outcome events or summary measures over time<br>*Case-control study* - Report numbers in each exposure	N/A for this study.		

		category, or summary measures of exposure<br>*Cross-sectional study* - Report numbers of outcome events or summary measures			
Main results	16	(a) Give unadjusted estimates and, if applicable, confounder-adjusted estimates and their precision (e.g., 95% confidence interval). Make clear which confounders were adjusted for and why they were included<br>(b) Report category boundaries when continuous variables were categorized<br>(c) If relevant, consider translating estimates of relative risk into absolute risk for a meaningful time period	Line 54-98 (following table 1)		
Other analyses	17	Report other analyses done—e.g., analyses of subgroups and interactions, and sensitivity analyses	N/A for this study.		
**Discussion**					
Key results	18	Summarise key results with reference to study objectives	Line 101, (following table 1)		
Limitations	19	Discuss limitations of the study, taking into account sources of potential bias or imprecision. Discuss both direction and magnitude of any potential bias	Line 170 (following table 1)	RECORD 19.1: Discuss the implications of using data that were not created or collected to answer the specific research question(s). Include discussion of misclassification bias, unmeasured confounding, missing data, and changing eligibility over time, as they pertain to the study being reported.	Line 170 (following table 1)
Interpretation	20	Give a cautious overall interpretation of results considering objectives, limitations, multiplicity of	Line 119-169 (following table 1)		

For peer review only

		analyses, results from similar studies, and other relevant evidence			
Generalisability	21	Discuss the generalisability (external validity) of the study results	Discussed throughout discussion section line 101-189 (following table 1)		
**Other Information**					
Funding	22	Give the source of funding and the role of the funders for the present study and, if applicable, for the original study on which the present article is based	Line 205 (following table 1)		
Accessibility of protocol, raw data, and programming code		..		RECORD 22.1: Authors should provide information on how to access any supplemental information such as the study protocol, raw data, or programming code.	Line 191 (following table 1)

*Reference: Benchimol EI, Smeeth L, Guttmann A, Harron K, Moher D, Petersen I, Sørensen HT, von Elm E, Langan SM, the RECORD Working Committee. The REporting of studies Conducted using Observational Routinely-collected health Data (RECORD) Statement. *PLoS Medicine* 2015; in press.

*Checklist is protected under Creative Commons Attribution (CC BY) license.

For peer review only

BMJ Open

Exploring emergency department four-hour target performance and cancelled elective operations. A regression analysis of routinely collected and openly reported NHS trust data.

Journal:	*BMJ Open*
Manuscript ID	bmjopen-2017-020296.R2
Article Type:	Research
Date Submitted by the Author:	15-Mar-2018
Complete List of Authors:	Keogh, Brad; University of Southampton Faculty of Health Sciences<br>Culliford, David; University of Southampton, Faculty of Health Sciences<br>Guerrero-Luduena, Richard; University of Southampton Faculty of Health Sciences<br>Monks, T; University of Southampton Faculty of Health Sciences
**Primary Subject Heading**:	Emergency medicine
Secondary Subject Heading:	Emergency medicine
Keywords:	4 hour target, performance target, Accident & Emergency, five year forward view

SCHOLARONE™
Manuscripts

Exploring emergency department four-hour target performance and cancelled elective operations. A regression analysis of routinely collected and openly reported NHS trust data.

Brad Keogh*, David Culliford, Richard Guerrero-Luduena, Thomas Monks

NIHR CLAHRC Wessex, University of Southampton, Building 67 E2005, Southampton, SO17 1BJ
Brad Keogh
Research Fellow

NIHR CLAHRC Wessex, University of Southampton, Building 67 E2005, Southampton, SO17 1BJ
David Culliford
Senior Medical Statistician

Centre for Implementation Science, University of Southampton, Building 67 E2007, Southampton, SO17 1BJ
Richard Guerrero-Luduena
Research Fellow

NIHR CLAHRC Wessex, University of Southampton, Building 67 E2005, Southampton, SO17 1BJ
Thomas Monks
Principal Research Fellow

* Correspondence to: brad.keogh@soton.ac.uk

For peer review only

For peer review only - http://bmjopen.bmj.com/site/about/guidelines.xhtml

What is already known on this topic

Pressure on emergency departments in England has increased steadily in recent years and adherence to the four-hour performance target has reduced. There is a lack of robust information around the causes of this decline. Calls from NHS England for trusts to return to previous performance levels are accompanied with suggestions of changes to services for which the impact is unclear and unevidenced.

What this study adds

Measures affecting patient flow throughout hospital systems including: admission pressures, hospital occupancy and delayed transfers of care were collated and investigated in England between 2012-2016. The relationships between these factors, four-hour target and cancelled elective operations were quantified. The results indicate some of the measures proposed by NHS England to reduce pressure on EDs may not have the desired impact on returning services to previous performance levels.

ABSTRACT

Objective To quantify the effect of intra-hospital patient flow on Emergency Department (ED) performance targets and indicate if the expectations set by the NHS England five year forward review are realistic in returning emergency services to previous performance levels.

Design Linear regression analysis of routinely reported trust activity and performance data using a series of cross-sectional studies.

Setting NHS trusts in England submitting routine nationally reported measures to NHS England.

Participants 142 acute non-specialist trusts operating in England between 2012 and 2016.

Main outcome measures The primary outcome measures were: proportion of four-hour waiting time breaches and cancelled elective operations.

Methods Univariate and multivariate linear regression models were used to show relationships between the outcome measures, and various measures of trust activity including: empty day-beds, empty night-beds, day-to-night bed ratio, ED conversion ratio and delayed transfers of care.

Results Univariate regression results using the outcome of four-hour breaches showed clear relationships with: empty night-beds and ED conversion ratio between 2012-2016. The day-to-night bed ratio showed an increasing ability to explain variation in performance between 2015-2016. Delayed transfers of care showed little evidence of an association. Multivariate model results indicated that the ability of patient flow variables to explain four-hour target performance had reduced between 2012-2016 (19% to 12%), and had increased in explaining cancelled elective operations (7% to 17%).

Conclusions The flow of patients through trusts is shown to influence ED performance, however performance has become less explainable by intra-trust patient flow between 2012 and 2016. Some commonly stated explanatory factors such as delayed transfers of care showed limited evidence of being related. The results indicate some of the measures proposed by NHS England to reduce pressure on EDs may not have the desired impact on returning services to previous performance levels.

For peer review only - http://bmjopen.bmj.com/site/about/guidelines.xhtml

Strengths and limitations of this study

- This study is the first to examine in detail the in-hospital factors that influence ED four-hour performance.
- We analyse the change in the importance of common explanatory theories of hospital flow bottlenecks across five years using recent openly published data.
- There are potentially some data quality issues around the variables used in the analysis which may influence the conclusions.
- Although a relatively simple methodology was used to ensure transparency of the study, it is possible that some statistical inference from the data is lost which more complex methodologies might reveal.
- Future work involving the use of more complex statistical methodologies and the investigation of the relative importance of: patient flows within trusts, population factors and data quality, would be of use to further understand the different pressures trusts face and aid in the targeting of service re-configurations.

For peer review only

For peer review only - http://bmjopen.bmj.com/site/about/guidelines.xhtml

INTRODUCTION

Background

It is widely reported that pressures on acute NHS trusts across England have been steadily increasing in recent years [1–5] . This is often reported in the media as rising numbers of breaches of the four-hour target, which is calculated as the percentage of patients being treated within four hours of arriving at an Emergency Department (ED). Concerns over the number of cancelled elective operations at trusts, as a result of increasing emergency pressures, have also been highlighted [5]. There is increasing pressure for NHS services to return to a 95% adherence of the four-hour target as part of the NHS England 'Next steps on the NHS five year forward view'[6], which has received some criticism [7]. Deliverables have been set which include looking to increase 'front door streaming', improving patient flow and reducing delayed transfers of care. There is currently uncertainty around the impact these interventions will have and if they are likely to result in a return to the performance targets expected.

There are several analyses which aim to understand the causes for ED performance decline and increasing pressure on acute services in England over recent years. Some analyses and commentary have suggested that high rates of bed occupancy and delayed transfers of care within trusts could be increasing the pressure on acute services, and in some cases may lead to increased waiting times in ED departments [3,8–11]. However, there is currently limited peer-reviewed statistical evidence showing the relationship between these factors and routinely collected measures of pressure on acute trusts. One of the few peer-reviewed works that has been published [12] examined data over a single two-week period in 2002 and used linear regression analysis to show a relationship between hospital bed occupancy and four-hour target performance. The study is univariate however and is limited to only a small time-window, hence it only provides a limited amount of understanding of the contributing factors to four-hour target performance. Other quantitative studies investigating the cases and consequences around waiting times in EDs [13–22] have looked at data which is not openly published and often pertains only to a small number or single hospital not necessarily located in the UK. Many of these studies also only look at the ED in isolation. These studies have limited scope in explaining the relative importance of different factors affecting growing emergency pressures on hospitals in England.

A systematic quantitative study is required which looks at the patient flow factors across trusts to understand the factors that trusts can modify which could improve performance and care. Greater understanding of the factors may allow appropriate targeting of resources tailored to trusts, rather than a suite of measures which are expected to be implemented across all providers. The aim of this work is to investigate the relative impact of the commonly highlighted variables: bed occupancy, delayed transfers of care, and other routinely measured operational factors, on the four-hour target as well as on cancelled elective operations across Acute trusts in England. This will provide evidence of the relative importance of each of these factors as well as how these dependencies have changed over time. It will also demonstrate how routinely collected and openly reported data can be utilised in a statistically robust way to understand more about how pressures on NHS services are changing.

Simplified high level system flow

Figure 1 shows a simplified hospital trust system and the patient flows through it. Patients attending ED will either be admitted as an inpatient to the trust or will be discharged from the ED. Patients may only be admitted from ED to the inpatient provision if there is space available (usually in the form of a bed). Space will only become available as inpatients are discharged or transferred from the trust to: home, social care provision or another trust. Additionally, there is pressure to admit from patients undergoing elective (non-emergency) procedures. Any of these patient flows could be a bottleneck which can result in a deterioration of trust performance measures.

Two measures of the pressure a trust is facing are breached attendances and cancelled elective operations; these are commonly thought to be a measure of pressure on EDs and inpatient provision respectively. There are several factors, related to patient flow, which may provide insight into the pressures that trusts are currently facing. Those which will be investigated in this study are related to: bed occupancy, which is reported both in day and night beds; day to night bed ratio, indicating the split between bed types in a trust; delayed transfers of care; conversion ratio, the proportion of patients attending ED who are admitted; casemix ratio, indicating the split between emergency

For peer review only - http://bmjopen.bmj.com/site/about/guidelines.xhtml

and elective admissions; and the number of admissions per bed within a trust. Figure 1 highlights these measures around the area they are likely to affect the hospital system most acutely.

Figure 1: a simplified trust system broadly illustrating patient flow.

For peer review only

Internal workings of an ED

The efficiency of internal ED micro-flow has been analysed extensively using modelling and simulation[23–25]. This, for example, has explored the use of fast tracks for minor injuries or patient 'streaming' (now common place in the England[26]; prioritisation by acuity[17]; and workforce scheduling and resourcing[27]. Processes of patient flow through ED and into trusts have also been shown to vary considerably between sites [17]. However, our unit of analysis is the hospital and its effects on four-hour performance.

METHODS

Data collation

A data set was collated from published open-data hosted on the NHS England Statistics website [28].These data sets included data for all NHS trusts in England, as well as minor injury units and walk-in-centres. At the time of collation data was available from quarter 2, 2011 until quarter 4, 2016 (calendar year) for the statistical reports entitled:

- Emergency department attendances and emergency admissions
- Bed availability and occupancy
- Cancelled elective operations
- Delayed transfers of care
- Hospital activity

The data source is created from each NHS organisation routinely reporting their own counts of activity, which NHS England collate and publish. This is a separate source from the Secondary Uses Service or Hospital Episodes Statistics. Full definitions of the indicators and the rules for submission by the providers are available from NHS England [28].

Trust filtering

For peer review only - http://bmjopen.bmj.com/site/about/guidelines.xhtml

The collated dataset was filtered to only include NHS trusts in England defined as: Small, Medium, Large Acute and Teaching. These trusts are the organisations who have seen the greatest reduction in the four-hour target. Mental health trusts, acute specialist trusts, walk in centres, practices, health centres, out-of-hours services and treatment centres were all excluded from the analysis. The definitions for NHS trust types are available online [29].

Study variables

The collated data contained counts of events for each NHS trust in England, which were converted into a proportion or a ratio using an appropriate denominator specific to the same trust for each time-period. For example: the ‘number of attendances in ED lasting greater than four-hours’ was divided by ‘total number of ED attendances’ of the period at that trust, and the ‘number of beds occupied’ were divided by ‘total number of beds’. This created variables, which allowed useful comparisons between trusts of different sizes and activity levels. A summary of the variables investigated in this study are included in table 1, along with information on how they were calculated and a description of how they should be interpreted. The variables in this study were created from aggregating quarterly data for each year between (2012-2016) and relate to those in figure 1. The raw collated quarterly count data as well as the python code to produce the year-aggregated variable data used in this study has also been made available [30].

For peer review only

Table 1: Variables included in the study.

Variables	Type	Numerator	Denominator	Units	Transformation applied?	Interpretation
Breached attendances	Outcome	Number of ED attendances greater than 4 hours	Number of ED attendances	-	-	Proportion of ED attendances waiting >4hrs.
Cancelled electives	Outcome	Number of cancelled elective operations	Number of elective admissions	Operations per admission	log	Ratio of cancelled elective operations to elective admissions. In absence of number of planned elective operations this is the most suitable denominator.
Empty day beds	Explanatory	Number of unoccupied day beds	Number of day beds	-	Categorised (5)	Ratio of unoccupied day beds to total number of day beds.
Empty night beds	Explanatory	Number of unoccupied night beds	Number of night beds	-	log	Ratio of unoccupied night beds to total number of night beds.
Delayed transfers	Explanatory	Number of bed days taken by delayed transfers	Number of night beds	10 bed-days	-	The number of bed-days lost to delayed transfers for each night bed at a trust, over the course of a year.
Day to night bed ratio	Explanatory	Number of day beds	Number of night beds	-	-	Ratio of total day beds to total night beds.
ED conversion ratio	Explanatory	Number of emergency admissions via ED	Number of attendances at ED	-	-	Ratio of ED admissions to attendances. Often commonly referred to as 'Conversion ratio'.
Admission casemix ratio	Explanatory	Number of non-elective (emergency) admissions	Number of elective admissions	-	log	Proportion of admissions that are emergency or ratio of emergency to elective admissions.
Emergency admission/ bed ratio	Explanatory	Number of non-elective (emergency) admissions	Number of day and night beds	10 Admissions	-	Number of emergency admissions per bed over the course of a year.

For peer review only - http://bmjopen.bmj.com/site/about/guidelines.xhtml

Variable distributions and transformation

Some variables were transformed to provide a normal distribution for regression model fitting. After conversion into proportions/ratios, non-normal variables were transformed using a natural log function (cancelled electives, empty night beds, admission casemix ratio). One variable (empty day beds) contained zero values and hence a log transformation was not appropriate. This variable was categorised. Bin edges were determined to provide approximately equal numbers of samples in each group. Where this was conducted bin sizes were created based on data across all years of study in order to provide consistent transformation.

Bias

Some missing data was found where trusts had not submitted data. For each year studied no more than 4% of trusts were found to have missing data for at least one variable; therefore the maximum percentage of missing data points for any regression was less than 4%. Our initial protocol intended to blind the variables throughout the analysis, however variables were un-blinded part way through the study as it was decided that greater contextual understanding of the problem was required to fully develop the analysis.

Within Trusts the reported activity is split into types 1,2 &3. Type 2 and type 3 EDs are defined by NHS England as: Minor Injury Units, Eye Casualties, Urgent Care Centres and Walk-In Centres. At some trusts these services are co-located at the same hospital site and so these could not be excluded from the analysis as the attendances contribute to hospital patient flow. It is possible that some four-hour target variation across Trusts may be due to larger volumes of patients attending type 2 & 3 units for minor injuries. As Trusts are often measured by their four-hour target performance based on all ED types, and the focus of this work is on patient flow across Trusts, the analysis conducted in this paper includes attendances at all types of department within each Trust.

Statistical methods

Univariate ordinary least squares linear regression was conducted using breached attendances as the outcome variable against each of the explanatory variables. To ascertain how the importance of each variable has changed over time the regression analysis was performed for each year separately, in a series of cross-sectional studies. As this method could present a statistical problem of multiple comparisons, undue emphasis was not placed on statistical significance tests. Results are presented as an exploratory study, showing the regression coefficients, associated confidence intervals and coefficient of determination values in each case. Only consistent associations which are of clinical importance are highlighted in the discussion. Multivariate regression was also performed to ascertain the relative importance of each predictor variable on breached attendances when combined into a single model. A model containing all predictor variables which have been highlighted of clinical importance, and those which showed considerable association strength to the outcome variable in the univariate regression analysis, was created to provide some understanding of the interaction between predictor variables. The univariate and multivariate models' residuals were checked visually for normality and homogeneity. Influential outliers with high leverage were also investigated using Cook's distance, but not removed. Abnormalities are reported in the results section. The same method of analysis was repeated using cancelled elective operations as the explanatory variable. Examples of the plots used to check the models are provided in Appendix A.

All the analysis conducted in this work was completed with the python language (version 3.6.0, www.python.org) using the: Statsmodels (version 0.8.0, www.statsmodels.org) and Pandas (version 0.19.2, pandas.pydata.org) libraries. This was done in an Anaconda environment (www.anaconda.com) utilising Jupyter notebooks (www.jupyter.org).

Patient and public involvement

There was no patient or public involvement in this study.

RESULTS

For peer review only - http://bmjopen.bmj.com/site/about/guidelines.xhtml

In 2012 there were 254 organisations in England reporting at least one of the variables investigated in this study (226 in 2016). Figure 2 shows the distribution of four-hour target breaches by organisation type. The acute and teaching trusts are observed to have higher proportions of breaches, with increases between 2012 and 2016. Most non-acute and specialist trusts still conform to the four-hour target in 2016. Hence the former group were the focus of this study. The number of trusts reduced to 142 when applying the criteria of the organisation type for the study (135 in 2016). Missing data reduced the number of trusts for some variables to 136 (131 in 2016).

Figure 2: the distribution of four-hour target breaches by organisation type for 2012 and 2016.

How variables have changed over time

Table 2 quantifies changes of each variable between 2012-2016. Between 2012 and 2016 the median values of breached attendances and cancelled elective operations has increased. The spread of the values has also increased in the time period in both cases, however more noticeably by over three times in the case of ‘breached attendances’. The most noticeable increases occurred between 2015 and 2016 in both cases. There has been a decline the proportion of empty night beds between 2015 and 2016 (it should be noted that this variable is the reverse of occupancy; ‘empty beds’ = 1 – ‘occupancy’). This reflects the historical reduction in night beds which has been noted elsewhere [10]. Between 2012 and 2016 there has been a noticeable increase in delayed transfers and emergency admission to bed ratios across trusts. Median bed-days and IQR lost due to delayed transfers has doubled over the period. Less prominent increases are observed in: empty day beds, day to night bed ratio and admission casemix ratio over this period. Although there are some fluctuations in ED conversion ratio, there is no overall change across the period.

Table 2: Changes in distribution of explanatory variables between 2012 and 2016.

Variable		2012	2013	2014	2015	2016
Breached attendances	median	0.05	0.05	0.06	0.07	0.13
	IQR	0.02	0.02	0.03	0.06	0.07
	5-95%	0.05	0.07	0.10	0.11	0.16
Cancelled electives	median	0.012	0.013	0.013	0.013	0.015
	IQR	0.007	0.008	0.010	0.010	0.011
	5-95%	0.019	0.019	0.020	0.021	0.026
Empty day beds	median	0.10	0.10	0.09	0.11	0.11
	IQR	0.17	0.17	0.17	0.17	0.21
	5-95%	0.38	0.37	0.43	0.40	0.37
Empty night beds	median	0.13	0.13	0.13	0.13	0.11
	IQR	0.08	0.08	0.09	0.08	0.07
	5-95%	0.16	0.17	0.18	0.18	0.14
Delayed transfers	median	0.73	0.77	0.91	1.04	1.36

For peer review only - http://bmjopen.bmj.com/site/about/guidelines.xhtml

	IQR	0.76	0.71	0.75	1.05	1.18
	5-95%	1.56	1.72	1.88	2.25	2.99
Day to night bed ratio	median	0.10	0.10	0.10	0.11	0.11
	IQR	0.07	0.07	0.07	0.08	0.08
	5-95%	0.14	0.17	0.17	0.18	0.17
ED conversion ratio	median	0.22	0.23	0.23	0.24	0.23
	IQR	0.09	0.09	0.10	0.09	0.08
	5-95%	0.19	0.19	0.19	0.19	0.19
Admission casemix ratio	median	1.15	1.13	1.16	1.17	1.19
	IQR	0.39	0.51	0.45	0.41	0.40
	5-95%	1.12	1.21	1.59	1.55	1.67
Emergency admission/ bed ratio	median	4.42	4.47	4.62	4.76	4.93
	IQR	0.94	1.10	1.05	0.99	1.08
	5-95%	2.43	2.45	2.61	2.65	2.69

Breached Attendances: Univariate regression analysis

Table 3 gives the results of the univariate regression models using breached attendances as the outcome variable. Night bed emptiness and ED conversion ratio showed respectively positive and negative associations, with breached attendances, consistently for each year of study. For night bed emptiness in 2012 the R^2 value 0.10 is of similar magnitude to a previous study [12], however in subsequent years is observed to reduced. The day to night bed ratio showed a negative relationship with trust breaches, which increased in strength during 2015-2016; it also shows an increasing ability to account for breaches (increasing R value between 2014-2016). The delayed transfers variable showed little evidence of a linear association with breached attendances between 2012-2015.

Table 3: univariate regression for the breached attendances outcome variable. Note: all results were based on data from 131 or more trusts (* indicates non-normality, heteroskedasticity or influential outlier in regression; 'Reg coef' is the regression coefficient, β).

Variable	Parameter	Year				
		2012	2013	2014	2015	2016
Empty day beds	R^2	0.02	0.01	0.01*	0.00*	0.00
	Reg coef (95% CIs)	-0.001 (-0.003,0.001)	-0.001 (-0.004,0.002)	0.001 (-0.002,0.005)	0.001 (-0.003,0.006)	0.002 (-0.004,0.008)
	p	0.15	0.43	0.4	0.54	0.6
Empty night beds	R^2	0.10*	0.09	0.07	0.05	0.06*
	Reg coef (95% CIs)	-0.012 (-0.019,-0.006)	-0.015 (-0.022,-0.007)	-0.017 (-0.027,-0.006)	-0.017 (-0.031,-0.004)	-0.025 (-0.042,-0.008)
	p	<0.01	<0.01	<0.01	0.01	<0.01
Delayed transfers	R^2	0.04*	0.02	0.01*	0.01	0.04
	Reg coef (95% CIs)	0.006 (0.001,0.011)	0.005 (-0.001,0.011)	0.003 (-0.004,0.011)	0.003 (-0.005,0.011)	0.011 (0.002,0.020)
	p	0.01	0.13	0.36	0.43	0.02
Day to night bed ratio	R^2	0.03	0.02	0.01	0.04	0.05
	Reg coef (95% CIs)	-0.058 (-0.118,0.002)	-0.066 (-0.138,0.006)	-0.058 (-0.156,0.040)	-0.146 (-0.264,-0.027)	-0.218 (-0.378,-0.058)
	p	0.06	0.07	0.25	0.02	0.01
ED conversion	R^2	0.07	0.05	0.05*	0.05	0.03
	Reg coef (95% CIs)	0.071 (0.027,0.115)	0.082 (0.025,0.140)	0.114 (0.033,0.195)	0.143 (0.039,0.248)	0.144 (0.000,0.288)

For peer review only - http://bmjopen.bmj.com/site/about/guidelines.xhtml

	p	<0.01	0.01	0.01	0.01	0.05
Admission casemix ratio	R^2	0.00	0.00	0.00	0.00*	0.00
	Reg coef (95% CIs)	0.002 (-0.007,0.010)	0.001 (-0.010,0.012)	-0.002 (-0.016,0.012)	0.007 (-0.012,0.025)	-0.001 (-0.027,0.024)
	p	0.70	0.86	0.77	0.47	0.93
Emergency admission to bed ratio	R^2	0.01	0.02	0.01*	0.04	0.01
	Reg coef (95% CIs)	0.002 (-0.001,0.006)	0.003 (-0.001,0.008)	0.003 (-0.002,0.009)	0.009 (0.001,0.017)	0.005 (-0.005,0.015)
	p	0.21	0.14	0.25	0.03	0.33

Breached attendances: Multivariate regression analysis

Table 4 shows the results of the multivariate regression for 2012 and 2016. In 2012 empty night beds and ED conversion ratio variables were both statistically significant and the model was able to explain 19% of the variation in the breached attendances variable. In 2016 the results of the multivariate model show only day to night bed ratio to be statistically significant in predicting breached attendances. The R^2 value indicates that only 12% of the variation in breached attendances can be accounted for with the parameters investigated in this study in 2016. When applying the multivariate model for other years a reduction in the importance of empty night beds and ED conversion ratio, and an increase in importance of day to night bed ratio was steadily observed between 2012-2016.

Table 4: multivariate regression model output for breached attendances as explanatory variable (R^2 = 0.19 in 2012, R^2 = 0.12 in 2016).

	2012			2016		
Variable	Regression coefficient	p	95% CIs	Regression coefficient	p	95% CIs
Empty night beds	-0.010	<0.01	-0.016 -0.004	-0.016	0.09	-0.034 0.003
Delayed transfers	0.004	0.12	-0.001 0.008	0.006	0.24	-0.004 0.015
Day to night bed ratio	-0.045	0.12	-0.102 0.012	-0.169	0.04	-0.330 -0.008
ED conversion ratio	0.068	<0.01	0.025 0.112	0.104	0.17	-0.044 0.252

Cancelled elective operations: Univariate regression analysis

Table 5 gives the results of the univariate regression models using cancelled elective operations as the outcome variable. ED conversion ratio, admission casemix ratio and delayed transfers showed a positive relationship with cancelled elective operations. Between 2014-2016 the variables ED conversion ratio and admission casemix ratio show increasingly positive associations with cancelled elective operations. These variables also demonstrate a relatively high ability to explain the outcome variable in comparison to the other variables in the study (R^2 values 0.07 & 0.11 respectively in 2016). The delayed transfers variable is observed to increase in importance between 2013-2016. The other variables included in the study did not show evidence of any clear association with cancelled elective operations.

Table 5: univariate regression output for the cancelled elective operations outcome variable (* indicates non-normality, heteroskedasticity or influential outlier in regression; 'Reg coef' is regression coefficient, β).

Variable	Parameters	Year				
		2012	2013	2014	2015	2016

For peer review only

Empty day beds	R^2	0.00	0.00	0.00	0.00	0.00
	Reg coef	0.009	0.009	0.008	-0.014	-0.003
	(95% CIs)	(-0.047,0.066)	(-0.046,0.064)	(-0.051,0.066)	(-0.079,0.051)	(-0.069,0.062)
	p	0.75	0.75	0.8	0.68	0.92
Empty night beds	R^2	0.01	0.02	0.00	0.00	0.02
	Reg coef	-0.135	-0.130	-0.087	-0.066	-0.149
	(95% CIs)	(-0.328,0.059)	(-0.307,0.047)	(-0.273,0.099)	(-0.260,0.129)	(-0.336,0.039)
	p	0.17	0.15	0.36	0.51	0.12
Delayed transfers	R^2	0.01	0.00*	0.01*	0.03*	0.03
	Reg coef	0.087	0.040	0.065	0.108	0.103
	(95% CIs)	(-0.064,0.237)	(-0.097,0.178)	(-0.063,0.192)	(-0.007,0.222)	(0.004,0.201)
	p	0.26	0.56	0.32	0.07	0.04
Day to night bed ratio	R^2	0.01	0.00	0.00	0.00	0.00
	Reg coef	1.067	0.589	0.245	0.255	-0.677
	(95% CIs)	(-0.733,2.868)	(-0.997,2.175)	(-1.410,1.900)	(-1.444,1.954)	(-2.437,1.082)
	p	0.24	0.46	0.77	0.77	0.45
ED conversion	R^2	0.05	0.02	0.07	0.06	0.07
	Reg coef	1.728	1.020	2.275	2.108	2.419
	(95% CIs)	(0.402,3.055)	(-0.288,2.327)	(0.914,3.637)	(0.627,3.589)	(0.901,3.937)
	p	0.01	0.13	<0.01	<0.01	<0.01
Admission casemix ratio	R^2	0.02	0.05	0.13	0.12*	0.11
	Reg coef	0.240	0.318	0.507	0.524	0.518
	(95% CIs)	(-0.016,0.496)	(0.082,0.553)	(0.288,0.726)	(0.275,0.772)	(0.258,0.778)
	p	0.07	<0.01	<0.001	<0.001	<0.001
Emergency admission to bed ratio	R^2	0.00	0.00	0.00	0.00	0.00
	Reg coef	0.029	0.010	0.009	-0.025	-0.033
	(95% CIs)	(-0.077,0.136)	(-0.087,0.108)	(-0.089,0.107)	(-0.136,0.087)	(-0.143,0.077)
	p	0.58	0.84	0.85	0.66	0.56

Cancelled electives: Multivariate regression analysis

Table 6 shows a summary of the variables included in the multivariate linear regression model using cancelled elective operations as an explanatory variable in 2012 and 2016. In 2012 the only statistically significant variable related to cancelled elective operations was ED conversion ratio, which had a positive association. In 2016 the results indicate that the statistically significant variables in explaining variation were ED conversion ratio and admission casemix ratio. Both of these variables were observed to be statistically significant in the model for year between 2014-2016. Overall the model was able to account for 17% of the variation in cancelled elective operations in 2016, which demonstrates an increasing ability to determine cancelled elective operations over the study period (increasing from 7% in 2012).

Table 6: multivariate regression model output for cancelled electives as explanatory variable (R^2 = 0.07 in 2012; R^2 = 0.17 in 2016).

	2012			2016		
Variable	Regression coefficient	p	95% CIs	Regression coefficient	p	95% CIs
Delayed transfers	0.067	0.37	-0.081 0.215	0.070	0.15	-0.025 0.164
ED conversion ratio	1.625	0.02	0.302 2.949	1.881	0.01	0.385 3.377
Admission	0.225	0.08	-0.027	0.483	<0.001	0.230

For peer review only

For peer review only - http://bmjopen.bmj.com/site/about/guidelines.xhtml

casemix ratio			0.477			0.736

DISCUSSION

Summary of findings

The intra-hospital patient flow variables with greatest association with better acute trust four-hour target performance in England were found to have varied over the period 2012-2016. The main variables of interest in explaining performance in 2012 were found to be the proportion of empty night beds and ED conversion ratio. These variables were seen to have reducing importance over the subsequent years in the multivariate model. The day to night bed ratio variable was observed to have increased in importance between 2012 and 2016, and in the years 2015-2016 it was the most important patient flow factor in explaining four-hour target performance in the multivariate model. The results also show that intra-hospital patient flow is only responsible for explaining some of the variation in four-hour target performance, and between 2012-2016 demonstrated a reducing ability to explain this variation (R^2 value of model reducing from 0.19 to 0.12). There was limited evidence of a clear association between delayed transfers of care and four-hour target performance, either in the univariate or multivariate model results.

The main intra-hospital patient flow variables associated with higher levels of cancelled elective operations at acute trusts between 2012-2016 were ED conversion ratio and the admission casemix ratio. Between 2012-2016 the importance of the admission casemix ratio is observed to have increased beyond that of ED conversion ratio. The ability of the multivariate model to explain variation in cancelled elective operations has increased between 2012 and 2016 (R^2 value of model increasing from 0.07 to 0.17), indicating that the emergency workload that trusts face are becoming an increasingly important factor in elective procedures being cancelled in England.

How important is intra-hospital flow to the four-hour target?

The ability of the multivariate model to explain only 12% of the variation in four-hour target in 2016 (which has reduced since 2012 from 17%) indicates that there are other factors that are increasingly important in determining four-hour performance. By comparison, another multivariate model could predict only 6.8% variation in four-hour target (using patient demographics and satisfaction with GP service rates) [22]. There are a number of factors which may affect ED processes and performance measures: macro or intra-hospital flow (i.e. patient flows across the whole trust, such as those investigated in this study); micro-flow factors within departments/wards (i.e. staffing[4,17], work flows[26,31], access to diagnostics[17]); population factors (i.e. age, sex, deprivation[22,32], access to GP/community/social care services[22,33]); noise (i.e. recording errors, reporting differences[10], 'gaming' of four-hour target[5,17]). If the mechanisms of pressures facing acute trusts are to be understood more fully, future work is required to quantify the relative effects of each of these factors on trust performance measures.

Bed occupancy

In our univariate analysis night-bed occupancy was consistently associated with 4-hr performance (2012-2016). This is a result consistent with queuing theory. That is, if trusts do not provide adequate bed 'buffer' capacity to cope with the peaks of emergency admission demand then ED performance will decline. As such trusts must continue to focus on innovations to reduce night-bed occupancy. Targets such as 85% occupancy have been proposed elsewhere [34] although this seems unrealistic given in 2012 and 2016 respectively only 39% and 25% of trusts attained this level of night-bed occupancy (or lower).

Day to night bed ratio and using day beds as 'buffers'

Our results show that that trusts with higher ratio of day to night bed capacity were more likely to have higher four-hour performance in 2016. It has been highlighted that admission and discharge patterns through trusts have peaks at different times of day [9,11]. Hence an explanation for this result is that day-bed capacity can be used flexibly as a temporary 'buffer' for patient admission whilst the discharge of patients from the trust catches up during the day.

For peer review only - http://bmjopen.bmj.com/site/about/guidelines.xhtml

Day beds are defined as consultant-led beds that are closed overnight [28], hence patients are not able to occupy them for long periods as in the case of night beds, and the occupancy of such wards/areas will be low at the beginning of each day to allow admissions. It is possible that more trusts are taking advantage of this flexibility as the day to night bed ratio of trusts in England has increased between 2012-2016 (see table 2). This may be in a response to trusts operating at greater levels of occupancy (also see table 2) towards the end of this period, and requiring a way of ensuring bed 'buffers' are available to allow patients to be admitted more promptly. We note that if occupancy can be reduced this may remove the need to use day beds as internal buffers.

Delayed transfers

Our expectations were that delayed transfers of care would be a strong predictor of four-hour performance. Our results do not support this commonly held assumption. This suggests that hospital initiatives to reduce delayed transfers of care may not yield the expected benefits for ED waiting times. We in no way suggest that initiatives to reduce delayed transfers are not worthwhile, as expedited transfers to appropriate care such as rehabilitation or social care have clear clinical and quality of life benefits for patients. We do note the strong association between in-patient bed occupancy (night-bed occupancy) and delayed transfers of care (Spearman's rank correlation in 2016 = -0.27, p = 0.002). However, it is clear that a focus on only delayed transfers will not reduce occupancy to a level that is sufficient to release the pressure on EDs; more holistic approaches to reducing bed occupancy may be required.

'Clinical streaming' and conversion ratio

'Clinical streaming' in ED aims to triage patients within 15 minutes of their arrival, and refer patients to other appropriate services [35]. This aims to: reduce the load on EDs by only treating patients who cannot be treated by other services, and provide better patient flow within EDs by prioritising different routes of care suited to patient needs. With regard to our study focussing on patient flow it is plausible that clinical streaming may affect ED conversion ratio as there is currently some limited evidence that earlier review by senior clinicians may reduce avoidable admissions [36]. Our results show that ED conversion ratio was important in explaining some variation in four-hour performance and cancelled elective operations, however conversion ratios of trusts in England have not changed noticeably between 2012 -2016 (see table 2) whilst some trusts are already known to have introduced clinical streaming [17]. It is currently unclear if there would be significant changes to trust conversion ratios with the proposed roll-out of clinical streaming [6] by NHS England. More research is needed to understand if clinical streaming impacts on patient flow in a positive manner.

Strengths and weaknesses of study

This study is the first to examine in detail the in-hospital factors that influence ED four-hour performance. We have analysed the change in the importance of common explanatory theories of hospital flow bottlenecks across five years using recent openly published data. We believe the study provides a simple and transparent analysis which can contribute to the discussion around the causes of decline in ED four-hour performance targets.

Trust four-hour target reporting is known to be affected by 'gaming' [5], which may introduce extra variability into the relationships under investigation. However, this is the measure which trusts are judged and funded, therefore it is argued that it is suitable for use within this study. The quality of data recording by trusts of the 'delayed transfers' variable has been reported to be questionable [10] and under-reported [37], however there is currently little published evidence around this issue. The data quality may explain the uncertainly in the importance of this variable over time in our analysis.

The R^2 values found, although higher than other multivariate models predicting the same outcome [22], indicate that there is limited ability of the models to predict the outcome variables. It is possible that more complex statistical methodologies could provide a greater predictive capability. For example, it is plausible that extreme values in some of the variables investigated may lead to changes in another, and otherwise may have little impact. This may, for example, explain our results for Delayed Transfers of Care. Hence one possible example could be the use of generalised additive models, which would be able to account for possible non-linear relationships between the rates and ratios investigated in this study. It is also possible there may be trusts where specific variables impact on

For peer review only - http://bmjopen.bmj.com/site/about/guidelines.xhtml

outcome measures but are not relevant to other trusts. It may be possible to use mixed models to investigate this further. A detailed longitudinal analysis could also be of benefit to provide greater understanding of the dependence of the variables over the study period. The data and open-source analysis code are supplied with this publication to allow the further development of this work and the open development of more complex models.

This study focusses on the macro-flow factors across trusts. Future work including the relative importance of the macro flow, micro flow, population factors and noise would be of value to assess the different pressures trusts face and aid in the targeting of service re-configurations.

FOOTNOTES

We thank the NHS England Statistics Unify2 team, for help in accessing and understanding the open data utilised in this study.

Transparency: The lead author (the manuscript's guarantor) affirms that the manuscript is an honest, accurate, and transparent account of the study being reported; that no important aspects of the study have been omitted; and that any discrepancies from the study as planned (and, if relevant, registered) have been explained. The authors attest that we have obtained appropriate permissions and paid any required fees for use of copyright protected materials.

Data sharing: Extra data can be accessed via the Dryad data repository at doi:10.5061/dryad.n71684c

Ethics: This study was reviewed by University of Southampton ERGO ethics committee (reference 25957).

Contributors: RG created the initial research question. BK contributed to the initial design of the work and was responsible for the protocol/ethics, data acquisition and collation, coding, analysis and interpretation of results. DC was responsible for the statistical methodology and interpretation of results. TM was involved throughout the study with guidance in the design, methodology and interpretation of results. All authors were involved in the development and review of the manuscript.

Competing interests: All authors have completed the ICMJE uniform disclosure form at www.icmje.org/coi_disclosure.pdf and declare: no support from any organisation for the submitted work; no financial relationships with any organisations that might have an interest in the submitted work in the previous three years; no other relationships or activities that could appear to have influenced the submitted work.

Funding: This article presents independent research funded by the National Institute for Health Research (NIHR) Collaboration for Leadership in Applied Health Research and Care (CLAHRC) Wessex. The views expressed in this publication are those of the author(s) and not necessarily those of the National Health Service, the NIHR, or the Department of Health.

Copyright: The Corresponding Author has the right to grant on behalf of all authors and does grant on behalf of all authors, an exclusive licence (or non exclusive for government employees) on a worldwide basis to the BMJ Publishing Group Ltd to permit this article (if accepted) to be published in BMJ editions and any other BMJPGL products and sublicences such use and exploit all subsidiary rights, as set out in our licence.

REFERENCES

1. Blunt I, Edwards N, Merry L. What's behind the A&E "Crisis"? The Nuffield Trust. 2015.

2. Appleby J. Nuffield Winter Insight Briefing 1: Winter beds pressures. The Nuffield Trust. 2016.

3. Fisher E, Dorning H. Winter pressures: what's going on behind the scenes ? The Nuffield Trust. 2016.

For peer review only

For peer review only - http://bmjopen.bmj.com/site/about/guidelines.xhtml

4. House of Commons Health Committee. Urgent and emergency services. 2013.

5. House of Commons Health Comittee. Winter pressure in accident and emergency departments. 2016.

6. NHS England. Next Steps on the NHS Five Year Forward View. 2017.

7. Oliver D. Reducing acute admissions? a timely reality check. BMJ. 2017;357:j2082.

8. Monitor. A&E delays : Why did patients wait longer last winter? 2015.

9. Karakusevic S. Understanding patient flow in hospitals. The Nuffield Trust. 2016.

10. British Medical Association. State of the health system Beds in the NHS: England. 2017.

11. Allder S, Silvester K, Walley P. Understanding the current state of patient flow in a hospital. Clin Med (Lond). 2010;10(5):441–4.

12. Cooke MW, Wilson S, Halsall J, Roalfe A. Total time in English accident and emergency departments is related to bed occupancy. Emerg Med J. 2004;21(5):575–6.

13. Mustafa F, Gilligan P, Obu D, O'Kelly P, O'Hea E, Lloyd C, et al. "Delayed discharges and boarders": a 2-year study of the relationship between patients experiencing delayed discharges from an acute hospital and boarding of admitted patients in a crowded ED. Emerg Med J. 2016;33:636–40.

14. Sun BC, Laurie A, Prewitt L, Fu R, Chang AM, Augustine J, et al. Risk-Adjusted Variation of Publicly Reported Emergency Department Timeliness Measures. Ann Emerg Med. 2016;67(4):509–516.e7.

15. Rathlev NK, Chessare J, Olshaker J, Obendorfer D, Mehta SD, Rothenhaus T, et al. Time Series Analysis of Variables Associated With Daily Mean Emergency Department Length of Stay. Ann Emerg Med. 2007;49(3):265–71.

16. Silvester K, Harriman P, Walley P, Burley G. Does process flow make a difference to mortality and cost? An observational study. Int J Health Care Qual Assur. 2014;27(7):616–32.

17. Pinkney J, Rance S, Benger J, Brant H, Joel-Edgar S, Swancutt D, et al. How can frontline expertise and new models of care best contribute to safely reducing avoidable acute admissions? A mixed-methods study of four acute hospitals. Health Services and Delivery Research. 2016;4(3).

18. Blom MC, Jonsson F, Landin-Olsson M, Ivarsson K. Associations between in-hospital bed occupancy and unplanned 72-h revisits to the emergency department: a register study. Int J Emerg Med. 2014;7:25.

19. Graham J, Aitken ME, Shirm S. Correlation of measures of patient acuity with measures of crowding in a pediatric emergency department. Pediatr Emerg Care. 2011;27(8):706–9.

20. Harris A, Sharma A. Access block and overcrowding in emergency departments: an empirical analysis. Emerg Med J. 2010;27(7):508–11.

21. Vermeulen MJ, Ray JG, Bell C, Cayen B, Stukel TA, Schull MJ. Disequilibrium Between Admitted and Discharged Hospitalized Patients Affects Emergency Department Length of Stay. Ann Emerg Med. 2009;54(6):794–804.

22. Blunt I. Focus on: A&E attendances. Quality Watch: The Health Foundation and the Nuffield Trust. 2014.

23. Mohiuddin S, Busby J, Savovi J, Richards A, Northstone K, Hollingworth W, et al. Patient flow within UK emergency departments: a systematic review of the use of computer simulation modelling methods. BMJ Open. 2017;7:e015007.

24. Saghafian S, Austin G, Traub SJ. Operations research/management contributions to emergency department patient flow optimization: Review and research prospects. IIE Trans Healthc Syst Eng. 2015;5:101–23.

25. Gul M, Guneri AF. A comprehensive review of emergency department simulation applications for normal and disaster conditions. Comput Ind Eng. 2015;83:327–44.

26. Saghafian S, Hopp WJ, Oyen MP Van, Desmond JS, Kronick SL. Patient Streaming as a Mechanism for

For peer review only

For peer review only - http://bmjopen.bmj.com/site/about/guidelines.xhtml

Improving Responsiveness in Emergency Departments. Oper Res. 2012;60(5):1080–97.

27. Duguay C, Chetouane F. Modeling and Improving Emergency Department Systems using Discrete Event Simulation. Simulation. 2007;83(4):311–20.

28. NHS England. NHS England Statistics [Internet]. 2017 [cited 2017 May 1]. Available from: https://www.england.nhs.uk/statistics/

29. NHS Improvement. NHS trust list [Internet]. 2017 [cited 2017 May 1]. Available from: http://www.nrls.npsa.nhs.uk/EasySiteWeb/GatewayLink.aspx?alId=135142

30. Keogh B, Culliford D, Guerrero-Luduena R, Monks T. NHS England routinely reported measures 2011-2016 [Internet]. University of Southampton [Dataset]. 2017. Available from: doi:10.5258/SOTON/D0218

31. Han JH, France DJ, Levin SR, Jones ID, Storrow AB, Aronsky D. The effect of physician triage on emergency department length of stay. J Emerg Med. 2010;39(2):227–33.

32. O'Cathain A, Knowles E, Maheswaran R, Pearson T, Turner J, Hirst E, et al. A system-wide approach to explaining variation in potentially avoidable emergency admissions: national ecological study. BMJ Qual Saf. 2014;23:47–55.

33. Flores-Mateo G, Violan-Fors C, Carrillo-Santisteve P, Peiro S, Argimon J-M. Effectiveness of organizational interventions to reduce emergency department utilization: A systematic review. PLoS One. 2012;7(5):1–7.

34. Bagust A, Place M, Posnett JW. Dynamics of bed use in accommodating emergency admissions: stochastic simulation model. BMJ. 1999;319:155–8.

35. NHS England. Clinical Streaming in the Accident and Emergency (A & E) Department [Internet]. 2017. Available from: https://www.england.nhs.uk/wp-content/uploads/2017/07/principles-for-clinical-streaming-ae-department.pdf

36. O'Cathain A, Knowles E, Turner J, Maheswaran R, Goodacre S, Hirst E, et al. Explaining variation in emergency admissions: a mixed-methods study of emergency and urgent care systems. Health Services and Delivery Research. 2014;2(48).

37. Nuffield Trust. What's behind delayed transfers of care? [Internet]. [cited 2017 Jan 1]. Available from: https://www.nuffieldtrust.org.uk/resource/what-s-behind-delayed-transfers-of-care#dtocs-the-tip-of-the-iceberg

Figure Legends

Figure 1: a simplified trust system broadly illustrating patient flow.

Figure 2: the distribution of four-hour target breaches by organisation type for 2012 and 2016.

For peer review only - http://bmjopen.bmj.com/site/about/guidelines.xhtml

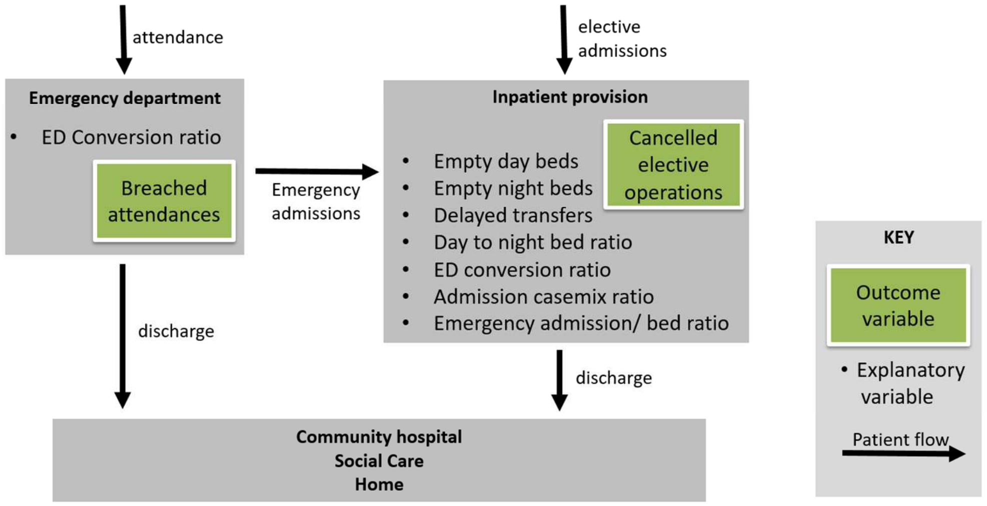

Figure 1 - a simplified trust system broadly illustrating patient flow

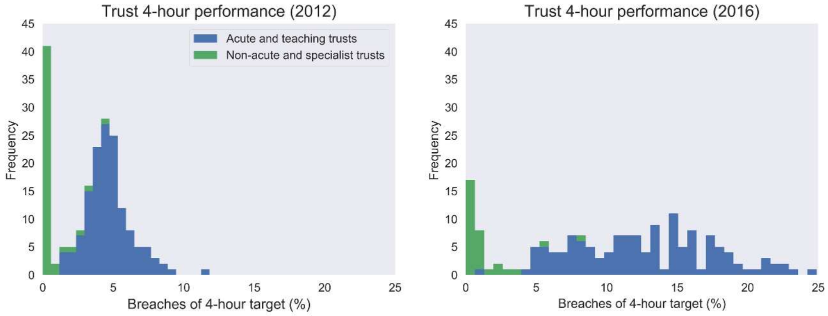

Figure 2 - the distribution of four-hour target breaches by organisation type for 2012 and 2016

For peer review only

Appendix A: Plotted outputs for sample regression case in 2016. All other models can be built, and similar plots produced, by using the data and code provided in the additional supplementary files.

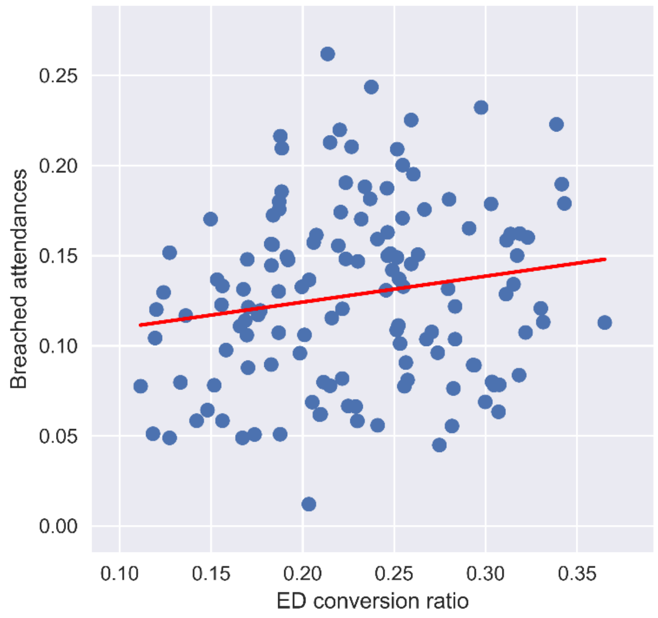

Figure A-1: Scatter plot of 'Breached attendances' and 'ED conversion ratio' with univariate linear regression model.

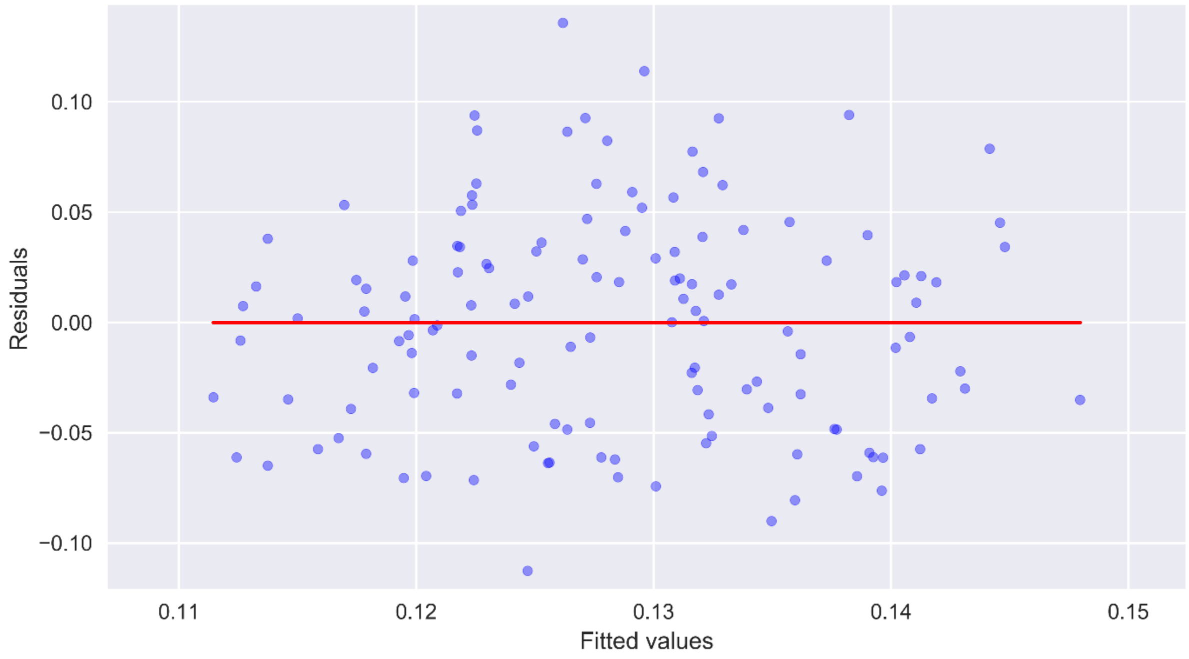

Figure A-2: Scatter plot of univariate regression residuals and fitted values from model in figure A-1.

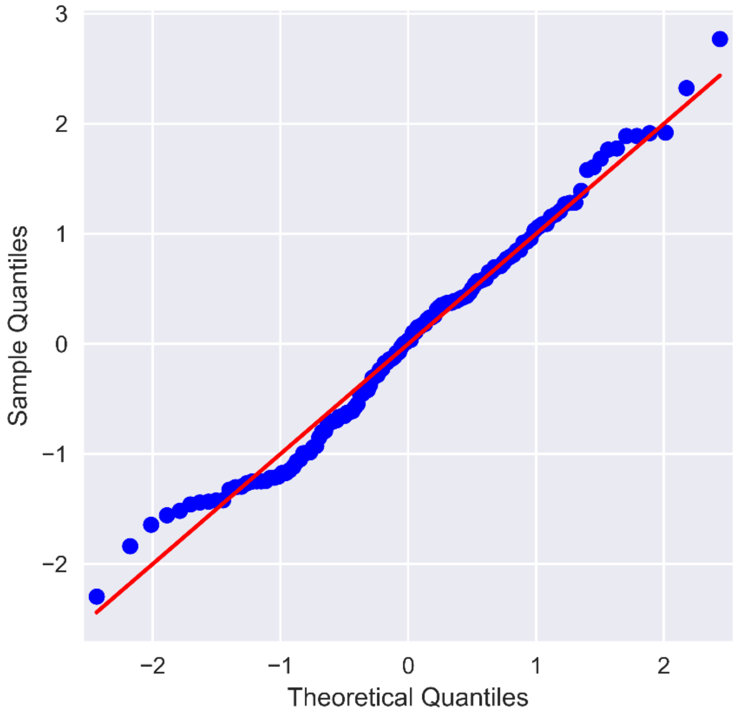

Figure A-3: QQ-plot of univariate regression model in figure A-1.

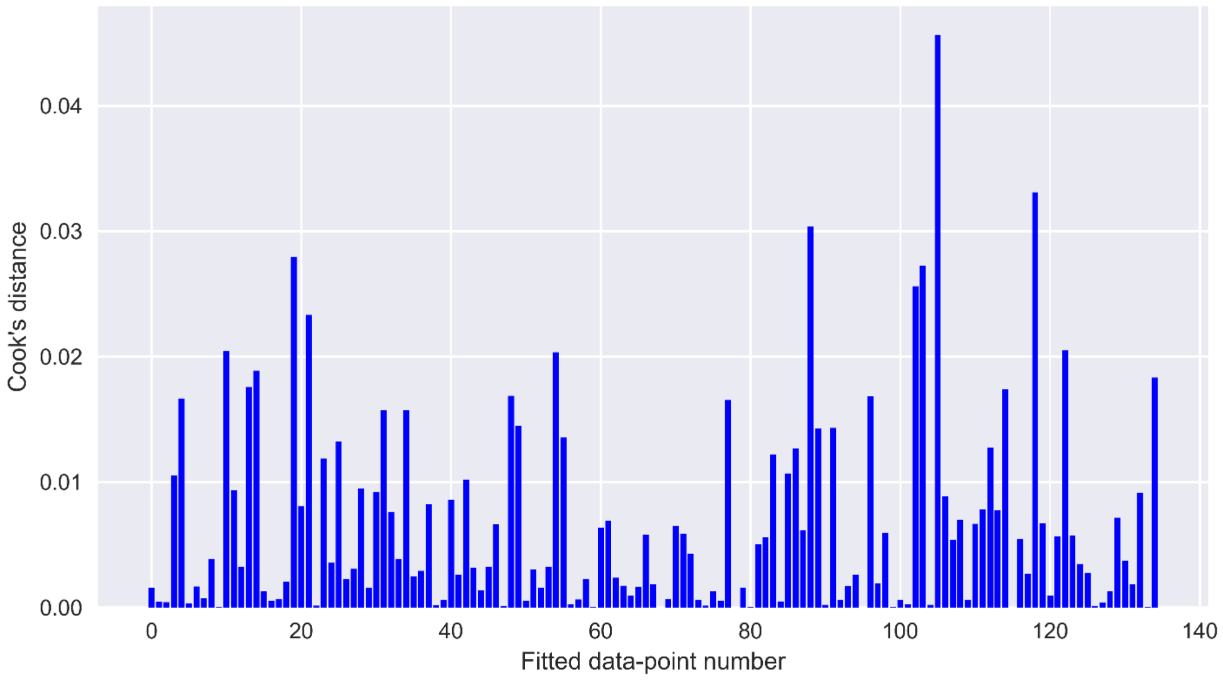

Figure A-4: Cook's distance values for each fitted data-point from model in figure A-1.

The RECORD statement – checklist of items, extended from the STROBE statement, that should be reported in observational studies using routinely collected health data.

Author note: this study is not a traditional observational study following patients (rather it follows NHS trusts), however the RECORD statement seemed most appropriate and helpful in reporting the study. As a result some of the RECORD statements were not applicable to this study. Most of the STROBE and RECORD items are located under the corresponding section titles in the manuscript but line/page numbers have been included below (all line numbers refer to authors' line numbering).

	Item No.	STROBE items	Location in manuscript where items are reported	RECORD items	Location in manuscript where items are reported
**Title and abstract**					
	1	(a) Indicate the study's design with a commonly used term in the title or the abstract (b) Provide in the abstract an informative and balanced summary of what was done and what was found	Pages 1 & 2	RECORD 1.1: The type of data used should be specified in the title or abstract. When possible, the name of the databases used should be included.<br><br>RECORD 1.2: If applicable, the geographic region and timeframe within which the study took place should be reported in the title or abstract.<br><br>RECORD 1.3: If linkage between databases was conducted for the study, this should be clearly stated in the title or abstract.	Page 2
**Introduction**					
Background rationale	2	Explain the scientific background and rationale for the investigation being reported	Page 3		
Objectives	3	State specific objectives, including any prespecified hypotheses	line 87		
**Methods**					
Study Design	4	Present key elements of study design early in the paper	See methods section starting line 121		

For peer review only

Setting	5	Describe the setting, locations, and relevant dates, including periods of recruitment, exposure, follow-up, and data collection	Line 123		
Participants	6	*(a) Cohort study* - Give the eligibility criteria, and the sources and methods of selection of participants. Describe methods of follow-up<br>*Case-control study* - Give the eligibility criteria, and the sources and methods of case ascertainment and control selection. Give the rationale for the choice of cases and controls<br>*Cross-sectional study* - Give the eligibility criteria, and the sources and methods of selection of participants<br><br>*(b) Cohort study* - For matched studies, give matching criteria and number of exposed and unexposed<br>*Case-control study* - For matched studies, give matching criteria and the number of controls per case	Line 137	RECORD 6.1: The methods of study population selection (such as codes or algorithms used to identify subjects) should be listed in detail. If this is not possible, an explanation should be provided.<br><br>RECORD 6.2: Any validation studies of the codes or algorithms used to select the population should be referenced. If validation was conducted for this study and not published elsewhere, detailed methods and results should be provided.<br><br>RECORD 6.3: If the study involved linkage of databases, consider use of a flow diagram or other graphical display to demonstrate the data linkage process, including the number of individuals with linked data at each stage.	6.1 included line 137.<br><br>6.2 & 6.3 not relevant for this study,
Variables	7	Clearly define all outcomes, exposures, predictors, potential confounders, and effect modifiers. Give diagnostic criteria, if applicable.	Table 1; page 5	RECORD 7.1: A complete list of codes and algorithms used to classify exposures, outcomes, confounders, and effect modifiers should be provided. If these cannot be reported, an explanation should be provided.	N/A for this study
Data sources/ measurement	8	For each variable of interest, give sources of data and details of methods of assessment (measurement).	Line 123		

For peer review only

		Describe comparability of assessment methods if there is more than one group			
Bias	9	Describe any efforts to address potential sources of bias	Line 7 (following table 1)		
Study size	10	Explain how the study size was arrived at	Line 30 (following table 1)		
Quantitative variables	11	Explain how quantitative variables were handled in the analyses. If applicable, describe which groupings were chosen, and why	Line 1 (following table 1)		
Statistical methods	12	(a) Describe all statistical methods, including those used to control for confounding<br>(b) Describe any methods used to examine subgroups and interactions<br>(c) Explain how missing data were addressed<br>(d) *Cohort study* - If applicable, explain how loss to follow-up was addressed<br>*Case-control study* - If applicable, explain how matching of cases and controls was addressed<br>*Cross-sectional study* - If applicable, describe analytical methods taking account of sampling strategy<br>(e) Describe any sensitivity analyses	Line 12 (following table 1)		
Data access and cleaning methods		..		RECORD 12.1: Authors should describe the extent to which the investigators had access to the database population used to create the study population.	Line 123<br><br><br><br>Line 1 (following

For peer review only - http://bmjopen.bmj.com/site/about/guidelines.xhtml

				RECORD 12.2: Authors should provide information on the data cleaning methods used in the study.	table 1)
Linkage		..		RECORD 12.3: State whether the study included person-level, institutional-level, or other data linkage across two or more databases. The methods of linkage and methods of linkage quality evaluation should be provided.	N/A for this study
**Results**					
Participants	13	(a) Report the numbers of individuals at each stage of the study (*e.g.*, numbers potentially eligible, examined for eligibility, confirmed eligible, included in the study, completing follow-up, and analysed)<br>(b) Give reasons for non-participation at each stage.<br>(c) Consider use of a flow diagram	Line 31 (following table 1)	RECORD 13.1: Describe in detail the selection of the persons included in the study (*i.e.,* study population selection) including filtering based on data quality, data availability and linkage. The selection of included persons can be described in the text and/or by means of the study flow diagram.	Line 31 (following table 1)
Descriptive data	14	(a) Give characteristics of study participants (*e.g.*, demographic, clinical, social) and information on exposures and potential confounders<br>(b) Indicate the number of participants with missing data for each variable of interest<br>(c) *Cohort study* - summarise follow-up time (*e.g.*, average and total amount)	N/A for this study, however descriptive data from line 40 (following table 1)		
Outcome data	15	*Cohort study* - Report numbers of outcome events or summary measures over time<br>*Case-control study* - Report numbers in each exposure	N/A for this study.		

For peer review only

		category, or summary measures of exposure<br>*Cross-sectional study* - Report numbers of outcome events or summary measures			
Main results	16	(a) Give unadjusted estimates and, if applicable, confounder-adjusted estimates and their precision (e.g., 95% confidence interval). Make clear which confounders were adjusted for and why they were included<br>(b) Report category boundaries when continuous variables were categorized<br>(c) If relevant, consider translating estimates of relative risk into absolute risk for a meaningful time period	Line 54-98 (following table 1)		
Other analyses	17	Report other analyses done—e.g., analyses of subgroups and interactions, and sensitivity analyses	N/A for this study.		
**Discussion**					
Key results	18	Summarise key results with reference to study objectives	Line 101, (following table 1)		
Limitations	19	Discuss limitations of the study, taking into account sources of potential bias or imprecision. Discuss both direction and magnitude of any potential bias	Line 170 (following table 1)	RECORD 19.1: Discuss the implications of using data that were not created or collected to answer the specific research question(s). Include discussion of misclassification bias, unmeasured confounding, missing data, and changing eligibility over time, as they pertain to the study being reported.	Line 170 (following table 1)
Interpretation	20	Give a cautious overall interpretation of results considering objectives, limitations, multiplicity of	Line 119-169 (following table 1)		

For peer review only

		analyses, results from similar studies, and other relevant evidence			
Generalisability	21	Discuss the generalisability (external validity) of the study results	Discussed throughout discussion section line 101-189 (following table 1)		
**Other Information**					
Funding	22	Give the source of funding and the role of the funders for the present study and, if applicable, for the original study on which the present article is based	Line 205 (following table 1)		
Accessibility of protocol, raw data, and programming code		..		RECORD 22.1: Authors should provide information on how to access any supplemental information such as the study protocol, raw data, or programming code.	Line 191 (following table 1)

*Reference: Benchimol EI, Smeeth L, Guttmann A, Harron K, Moher D, Petersen I, Sørensen HT, von Elm E, Langan SM, the RECORD Working Committee. The REporting of studies Conducted using Observational Routinely-collected health Data (RECORD) Statement. *PLoS Medicine* 2015; in press.

*Checklist is protected under Creative Commons Attribution (CC BY) license.

For peer review only
